# Chronic kidney disease in older adults: trends in prevalence and healthcare service quality from 2012 to 2018

**DOI:** 10.1093/ckj/sfaf180

**Published:** 2025-06-14

**Authors:** Tim Bothe, Elke Schaeffner, Anna Pöhlmann, Anne-Katrin Fietz, Julia Freitag, Nina Mielke, Jean-François Chenot, Muhammad Helmi Barghouth, Elizabeth Mathias, Simone Kiel, Natalie Ebert

**Affiliations:** Institute of Public Health, Charité – Universitätsmedizin Berlin, Berlin, Germany; Institute of Public Health, Charité – Universitätsmedizin Berlin, Berlin, Germany; Institute of Biometry and Clinical Epidemiology, Charité – Universitätsmedizin Berlin, Berlin, Germany; Institute of Biometry and Clinical Epidemiology, Charité – Universitätsmedizin Berlin, Berlin, Germany; AOK Nordost – Die Gesundheitskasse, Potsdam, Germany; Institute of Public Health, Charité – Universitätsmedizin Berlin, Berlin, Germany; Institute for Community Medicine, Department of General Practice, University Medicine Greifswald, Greifswald, Germany; Institute of Public Health, Charité – Universitätsmedizin Berlin, Berlin, Germany; Institute for Community Medicine, Department of General Practice, University Medicine Greifswald, Greifswald, Germany; Institute for Community Medicine, Department of General Practice, University Medicine Greifswald, Greifswald, Germany; Institute of Public Health, Charité – Universitätsmedizin Berlin, Berlin, Germany

**Keywords:** CKD, health claims data, incidence, prevalence, quality indicators

## Abstract

**Background:**

Data on the healthcare service quality for patients with chronic kidney disease (CKD) are vital for guiding practitioners, patients and healthcare policy makers. We examined new quality indicators for outpatient diagnostics and treatments in older patients with CKD, focusing on trends between 2012 and 2018.

**Methods:**

The study included cross-sectional German statutory health insurance claims data from four independent random samples (2012, 2014, 2016, 2018), each with 62 200 individuals aged ≥70 years. We analyzed coded CKD prevalence and incidence, non-recommended drug prescriptions (dual prescriptions of angiotensin-converting enzyme inhibitors with angiotensin II receptor blockers; non-steroidal anti-inflammatory drugs in CKD stage 4–5), as well as albumin/creatinine ratio (ACR) and dipstick testing in incident CKD cases.

**Results:**

After standardization, the samples included 58.4%–59.3% females, and mean ages ranging from 77.4 to 78.9 years. CKD prevalence increased from 17.8% [95% confidence interval (CI) 17.5; 18.1] in 2012 to 25.7% (95% CI 25.4; 26.1) in 2018. CKD incidence rose slightly from 6.4% (95% CI 6.2; 6.6) to 7.6% (95% CI 7.4; 7.9). Non-recommended drug prescriptions, which were below 5% in 2012, decreased by more than half by 2018. ACR and dipstick testing varied inconsistently over time, ranging from 11.4% to 13.5% and 55.4% to 57.2%, respectively.

**Conclusions:**

CKD prevalence in older adults in Germany rose by eight percentage points from 2012 to 2018 while prescriptions of non-recommended drugs decreased in patients with CKD, indicating better diagnosis and guideline adherence. However, ACR and dipstick diagnostic was alarmingly low, and remained below recommended levels outside kidney specialist care, showing areas for improvement.

## Graphical Abstract

**Graphical Abstract:**
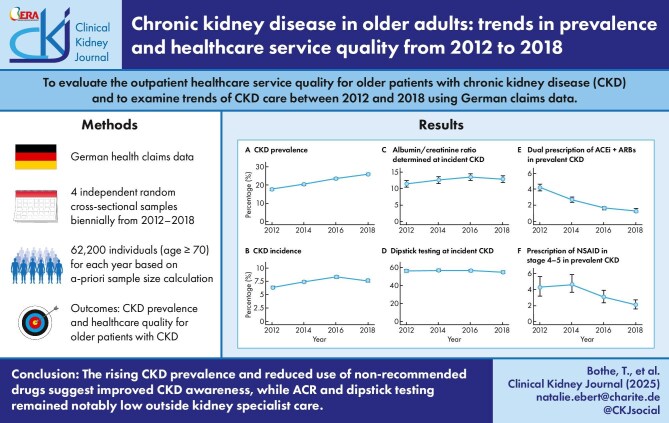

KEY LEARNING POINTS **What was known:**

- There is limited data on the outpatient healthcare service quality for older patients with chronic kidney disease (CKD) in Germany.
- Healthcare quality for CKD patients in Germany during the decade following the introduction of the Kidney Disease: Improving Global Outcomes (KDIGO) guidelines in 2012 has not been explored as yet.

**This study adds:**

- We examined newly developed quality indicators for diagnostics and treatments in older patients with CKD using biennial random cross-sectional German outpatient claims data from 2012 to 2018.
- We observed an increase in coded CKD prevalence and a decrease in non-recommended drug prescriptions in patients with CKD over time.
- Urine dipstick and albumin/creatinine ratio (ACR) testing outside the kidney specialist care were lower than expected and remained consistently low over the years.

**Potential impact:**

- This study offers a comprehensive analysis and evaluation of quality indicators for older patients with CKD.
- Over time, CKD prevalence in older adults in Germany rose by eight percentage points from 2012 to 2018 while prescriptions of non-recommended drugs decreased in patients with CKD, indicating better quality of care.
- The study underscores the need for greater disease awareness and more consistent ACR and urine dipstick testing in patients with CKD, particularly those at risk of CKD progression.

## INTRODUCTION

Raising disease awareness and optimizing the detection and diagnosis of chronic kidney disease (CKD) are crucial for timely treatment and preventive measures targeting disease progression [1–3]. This is particularly challenging due to the asymptomatic nature of CKD. In older patients where multiple comorbidities often coincide [4], detection of CKD has important implications for treatment decisions and drug dose adjustments, particularly when kidney function is severely impaired [5]. Also, the risks for adverse outcomes such as cardiovascular events or progression to chronic kidney failure (CKF) and mortality are known to be higher at more severe stages of CKD [6, 7].

CKD prevalence increases as a result of ageing populations in most industrialized regions, indicating a growing relevance of CKD [8, 9]. As shown in international data, CKD already affects a considerable proportion of older adults with higher prevalence estimates than in the general population [10]. Although referral rates to nephrologists and monitoring of proteinuria remain below recommended levels in Germany [11], other aspects of healthcare service quality for older patients with CKD have not been comprehensively evaluated [12], partly due to limited data and lack of accessible electronic claims databases.

The Kidney Disease: Improving Global Outcomes (KDIGO) guidelines were implemented globally in 2012 [13], yet it remains unclear whether CKD diagnostics and treatment quality of older patients have improved. Early diagnosis and effective treatment is particularly important for high-risk patients such as those with coexisting hypertension and diabetes mellitus, as it can reduce cardiovascular risk, slow CKD progression, and delay or prevent dialysis [14].

Using a multidisciplinary approach, we previously developed quality indicators (QI) for use in claims data to assess outpatient healthcare quality for older patients with non-dialysis-dependent CKD [12]. In Germany, approximately 87% of the population is covered by statutory health insurance [15], providing comprehensive coverage of healthcare services related to CKD.

In the present study we aimed to investigate trends over time in the quality of outpatient healthcare services for older patients with CKD by applying those QIs to German claims data. We hypothesized that CKD diagnosis and adherence to treatment guidelines improved following the implementation of the KDIGO guidelines in 2012.

## MATERIALS AND METHODS

### Study population, design and data source

As part of the project GUIDAGE-CKD (Guideline-compliant care of older patients with chronic kidney disease; funded by the Federal Joint Committees innovation fund; 01VSF20020), we investigated trends in the outpatient healthcare service quality for older patients with CKD using four independent random samples of claims data from 2012, 2014, 2016 and 2018. Each of the four samples consisted of 62 200 persons ≥70 years living in Northeast-Germany, all insured at the AOK Nordost—Die Gesundheitskasse which covers the largest share of older insurants in Northeast-Germany [16]. Data on sociodemographic information, outpatient treatments and services with corresponding diagnoses [International Classification of Diseases, 10th Revision, German Modification (ICD-10-GM)] [17], and prescribed and dispensed drugs were available. Single services were assessed using the German fee schedule for physicians (Gebührenordnungsposition; GOP) for outpatient services, operation and procedural (OPS) codes for inpatient services and anatomical therapeutic chemical (ATC) codes for drugs.

### Sample size calculation

We conducted a sample size calculation for the primary endpoint CKD prevalence and aimed to test six CKD-related predefined QI in total [12]. Based on the current literature, we estimated CKD prevalence in individuals aged ≥70 years at 25% in 2012 and assumed an increase up to 28% in 2018 [8, 18]. The sample size for each year tranche was calculated to be 6220 persons based on a *χ*²-test for Multiple Proportions, with a power of 80% and significance level of $0.008\bar{3}$ after Bonferroni-correction for six parallel hypotheses ($\alpha = \frac{{0.05}}{6}$). We accounted for all analyses to be stratified by sex and five age groups (70–74, 75–79, 80–84, 85–89 and ≥90 years), resulting in a final sample size of 62 200 persons per year tranche. The sample size was calculated using nQuery 8 (Statistical Solutions Ltd, Cork, Ireland).

### Selection criteria and sampling

The random sampling was conducted independently for each year tranche and stratified by sex and age. Persons with a history of kidney transplantation (ICD-10 Z94.0 or OPS 5-555 in 2006 through the year preceding a respective year tranche) were excluded before sampling. Patients with CKF treated with dialysis (ICD-10 Z49.1-2 or Z99.2 and GOP 13610–1 or OPS 8-853-7) were excluded after sampling [19].

### Quality indicators and operationalization within claims data

We assessed a total of six QI [12]. (i) CKD prevalence was defined as all diagnosed and coded cases with an estimated glomerular filtration rate (eGFR) <60 mL/min/1.73 m² identified using outpatient and inpatient diagnoses within a respective year tranche (ICD-10 N18.3-5, N18.8-9 and N19) [20]. (ii) CKD incidence was determined as all newly diagnosed and coded cases over all persons at risk, i.e. prevalent cases over all who had no respective diagnosis (ICD-10 N18.3-5, N18.8-9 or N19) in the year preceding the respective year tranche. We investigated differential diagnostic procedures as the proportion of incident CKD patients who had undergone outpatient (iii) urinary albumin/creatinine ratio (ACR) testing (QI_ACR_) and (iv) urine dipstick testing for protein (QI_Dipstick_). Finally, we analyzed non-recommended drug prescriptions as the proportion of patients with prevalent CKD and outpatient (v) dual prescription of angiotensin-converting enzyme (ACE) inhibitors and angiotensin II receptor blockers (ARBs) over several quarters of a year (QI_ACEi+ARB_) and the proportion of patients with CKD stage 4–5 for whom (vi) nonsteroidal anti-inflammatory drugs (NSAIDs) were prescribed (QI_NSAID_). In addition to NSAID prescription over time, we exploratorily assessed the proportion of patients with a prescription of the drug metamizole, an alternative non-NSAID analgesic without a contraindication in patients with severely reduced kidney function, hypothesizing that prescriptions of metamizole would increase if primary care physicians discontinued NSAID treatment.

For CKD prevalence and incidence, we further stratified into CKD stages 3 (eGFR 30–59 mL/min/1.73 m²) and 4–5 (eGFR <30 mL/min/1.73 m²) [13] using the stage-specific diagnoses (ICD-10 N18.3 and N18.4-5) [20]. We also identified the number of patients who received only stage-unspecific diagnoses (N18.8-9, N19). A list of the computational criteria and selection of diagnosis and service codes for each QI is provided in the Supplementary data, Table S1.

### Ethics statement

This study was approved by the local ethics committee at Charité—Universitätsmedizin Berlin (EA1/050/22).

### Statistical analysis

The main analysis was conducted overall for each year tranche. To account for sociodemographic differences due to the sampling procedure, all measures were standardized for the Northeast-German population using year-, sex- and age group-specific weights for Northeast-German states (Berlin, Brandenburg, Mecklenburg-Vorpommern) from the Federal Statistical Office (see Supplementary data, Table S2). The 95% confidence intervals (CIs) were calculated for all standardized QI. Time trends from 2012 through 2018 were tested with the Cochran–Armitage trend test [21, 22] after Bonferroni correction.

All analyses were stratified by sex and age, comorbidities (diabetes mellitus, arterial hypertension, both or neither), and regions of residence categorized as urban, semi-urban and rural. Diabetes and arterial hypertension were defined based on ICD-10 diagnoses as well as antidiabetic and antihypertensive medication intake. We additionally stratified all analyses by healthcare service provider groups [kidney specialists, general practitioners (GPs), hospitals and others]. “Kidney specialists” refers to both nephrology and urology outpatient care. All used definitions and computational criteria are described in Supplementary data, Tables S3 and S4. All statistical analyses were conducted with R (Version 4.2.2; R Foundation for Statistical Computing, Vienna, Austria).

## RESULTS

### Sociodemographic and clinical characteristics

In total, the four AOK claims data analysis samples consisted of 61 970 to 62 018 persons aged ≥70 years per year tranche, after 182–230 persons were excluded due to dialysis-dependent CKF (Table 1). The mean age increased from 77.4 years [standard deviation (SD) 5.9] in 2012 to 78.9 (SD 5.9) in 2018 and 58.4%–59.3% were female. Slightly less than half of individuals lived in urban areas and about a quarter each in semi-urban and rural areas. The prevalence of comorbidities varied slightly over the years with 34.5%–36.8% who had both diabetes and hypertension, 41.8%–42.6% who had arterial hypertension only and 4.0%–4.2% who had diabetes only.

**Table 1:** Sociodemographic and clinical characteristics for each year tranche.

	2012	2014	2016	2018
Total sample, *N*	62 200	62 200	62 200	62 200
Excluded patients with chronic dialysis*, n* (%)	230 (0.4)	214 (0.3)	182 (0.3)	206 (0.3)
Final analysis sample*, n* (%)	61 970 (99.6)	61 986 (99.7)	62 018 (99.7)	61 994 (99.7)
Demographics^a^				
Females, *n* (%)	36 758 (59.3)	36 423 (58.8)	36 348 (58.6)	36 210 (58.4)
Age (years), mean (SD)	77.4 (5.9)	77.8 (5.8)	78.5 (5.8)	78.9 (5.9)
Age groups (years), *n* (%)				
70–74	25 090 (40.5)	22 541 (36.4)	17 770 (28.7)	16 034 (25.9)
75–79	18 146 (29.3)	19 757 (31.9)	21 620 (34.9)	20 806 (33.6)
80–84	10 554 (17.0)	10 971 (17.7)	12 993 (21.0)	15 105 (24.4)
85–89	5500 (8.9)	5969 (9.6)	6547 (10.6)	6704 (10.8)
≥90	2680 (4.3)	2748 (4.4)	3088 (5.0)	3345 (5.4)
Region of residence^a^, *n* (%)				
Urban	27 764 (44.8)	27 976 (45.1)	28 478 (45.9)	28 618 (46.2)
Semi-urban	17 404 (28.1)	17 273 (27.9)	17 208 (27.7)	17 213 (27.8)
Rural	16 802 (27.1)	16 737 (27.0)	16 332 (26.3)	16 163 (26.1)
Comorbidities^a^, *n* (%)				
Arterial hypertension	47 775 (77.1)	48 364 (78.0)	48 654 (78.5)	48 679 (78.5)
Diabetes mellitus	23 986 (38.7)	24 582 (39.7)	25 292 (40.8)	25 290 (40.8)
Both diabetes mellitus and arterial hypertension	21 369 (34.5)	22 112 (35.7)	22 740 (36.7)	22 796 (36.8)
Only arterial hypertension	26 406 (42.6)	26 252 (42.4)	25 914 (41.8)	25 883 (41.8)
Only diabetes mellitus	2617 (4.2)	2470 (4.0)	2552 (4.1)	2494 (4.0)
Neither of the above	11 578 (18.7)	11 153 (18.0)	10 812 (17.4)	10 821 (17.5)

a The respective variables were standardized using year-, sex- and age group-specific weights for the population aged ≥70 years in Northeast-Germany.

### CKD prevalence and incidence

In 2012, coded CKD prevalence was 17.8% (95% CI 17.5; 18.1) and increased constantly to 25.7% in 2018 (95% CI 25.4; 26.1). There was a slight increase of coded CKD incidence from 6.4% (95% CI 6.2; 6.6) in 2012 to 7.6% (95% CI 7.4; 7.9) in 2018 while persons at risk for newly diagnosed CKD decreased from 50 032 to 44 903 (Table 2). Stage-specific analysis showed that the prevalence of CKD stage 3 increased from 8.2% to 15.7% and for CKD stage 4–5 from 2.8% to 4.4% (Supplementary data, Table S5). Meanwhile, the proportion of patients with exclusively stage-unspecific CKD diagnoses decreased from 6.7% in 2012 to 5.7% in 2018 (Supplementary data, Table S6 and Fig. S1).

**Table 2:** Prevalence, incidence and QIs of outpatient healthcare service quality for CKD with trends over time.

					Trend^c^	
	2012	2014	2016	2018	Δ	*P*
Prevalence: proportion of patients ≥70 years with diagnosed CKD						
Persons at risk^a^, *n*	61 970	61 986	62 018	61 994		
Proportion^b^, % (95% CI)	17.8% (17.5; 18.1)	20.5% (20.2; 20.8)	23.5% (23.1; 23.8)	25.7% (25.4; 26.1)	+8.0	<.0001^*^
Incidence: proportion of patients ≥70 years with newly diagnosed CKD						
Persons at risk^a^, *n* (%)	50 032 (80.7)	48 378 (78.0)	46 731 (75.4)	44 903 (72.4)		
Proportion^b^, % (95% CI)	6.4% (6.2; 6.6)	7.4% (7.2; 7.6)	8.3% (8.0; 8.5)	7.6% (7.4; 7.9)	+1.3	<.0002^*^
QI_ACR_: proportion of patients ≥70 years with incident CKD who had a urinary albumin/creatinine ratio determined						
Persons at risk^a^, *n* (%)	4258 (6.9)	4567 (7.4)	4644 (7.5)	4053 (6.5)		
Proportion^b^, % (95% CI)	11.4% (10.5; 12.4)	12.6% (11.6; 13.6)	13.5% (12.5; 14.5)	12.9% (11.9; 14.0)	+1.5	.0075
QI_Dipstick_: proportion of patients ≥70 years with incident CKD who had a dipstick-test for protein, glucose, erythrocytes, leukocytes and nitrite						
Persons at risk^a^, *n* (%)	4258 (6.9)	4567 (7.4)	4644 (7.5)	4053 (6.5)		
Proportion^b^, % (95% CI)	56.6% (55.1; 58.1)	57.2% (55.8; 58.6)	57.0% (55.6; 58.5)	55.4% (53.8; 56.9)	–1.2	.9949
QI_ACEi+ARB_: proportion of patients ≥70 years with CKD who had multiple (≥2) dual prescriptions of ACE inhibitors and ARBs over at least two quarters within three quarters after the quarter with the first simultaneous prescription (non-recommended)						
Persons at risk^a^, *n* (%)	8221 (13.3)	9424 (15.2)	10 763 (17.4)	12 058 (19.5)		
Proportion^b^, % (95% CI)	4.2% (3.8; 4.7)	2.7% (2.4; 3.1)	1.7% (1.5; 1.9)	1.3% (1.2; 1.6)	–2.9	<.0001^*^
QI_NSAID_: proportion of patients ≥70 years with CKD stage 4–5 (eGFR <30 mL/min/1.73 m²) for whom NSAIDs were prescribed (non-recommended)						
Persons at risk^a^, *n* (%)	1139 (1.8)	1626 (2.6)	1956 (3.2)	2234 (3.6)		
Proportion^b^, % (95% CI)	4.3% (3.2; 5.6)	4.6% (3.7; 5.8)	3.1% (2.4; 3.9)	2.1% (1.6; 2.8)	–2.2	<.0001^*^
Metamizole: proportion of patients ≥70 years with CKD stage 4–5 (eGFR <30 mL/min/1.73 m²) for whom metamizole was prescribed (exploratory)						
Persons at risk^a^, *n* (%)	1139 (1.8)	1626 (2.6)	1956 (3.2)	2234 (3.6)		
Proportion^b^, % (95% CI)	37.3% (34.5; 40.1)	43.8% (41.4; 46.2)	45.1% (42.9; 47.3)	45.7% (43.6; 47.7)	+8.4	n.a.

a The number of persons at risk indicates how many persons were eligible for testing of the respective QI (denominator), e.g. for incidence: only persons who did not have a CKD diagnosis in the respective previous year.

b The proportions for all QI were standardized using sex-, age group- and year-specific weights for the population aged ≥70 years in Northeast-Germany.

c Time trends were tested using the Cochran–Armitage trend test with Bonferroni correction.

^*^Statistically significant for *P* < .0083 (after Bonferroni correction).

Δ, difference from 2012 to 2018.

### Quality indicators for healthcare services in patients with CKD

The proportion of patients with incident CKD who had urine ACR determined (QI_ACR_) was generally very low and ranged from 11.4% (95% CI 10.5; 12.4) in 2012 to 12.9% (95% CI 11.9; 14.0) in 2018. Dipstick testing (QI_Dipstick_) was performed in 56.6% (95% CI 55.1; 58.1) of incident CKD patients in 2012 and 55.4% (95% CI 53.8; 56.9) in 2018 without any trend (Table 2).

The proportion of prevalent CKD patients who had dual prescriptions of ACE inhibitors and ARBs (QI_ACEi+ARB_) was 4.2% (95% CI 3.8; 4.7) in 2012 and declined to 1.3% (95% CI 1.2; 1.6) in 2018. During the same period, the proportion of NSAID prescriptions in patients with CKD stage 4–5 (QI_NSAID_) decreased from 4.3% (95% CI 3.2; 5.6) to 2.1% (95% CI 1.6; 2.8). While NSAIDs were prescribed less frequently over time, the proportion of patients with a prescription of the non-NSAID analgesic metamizole increased from 37.3% in 2012 to 45.7% in 2018.

### Subgroup and sensitivity analyses

**Figure 1:**
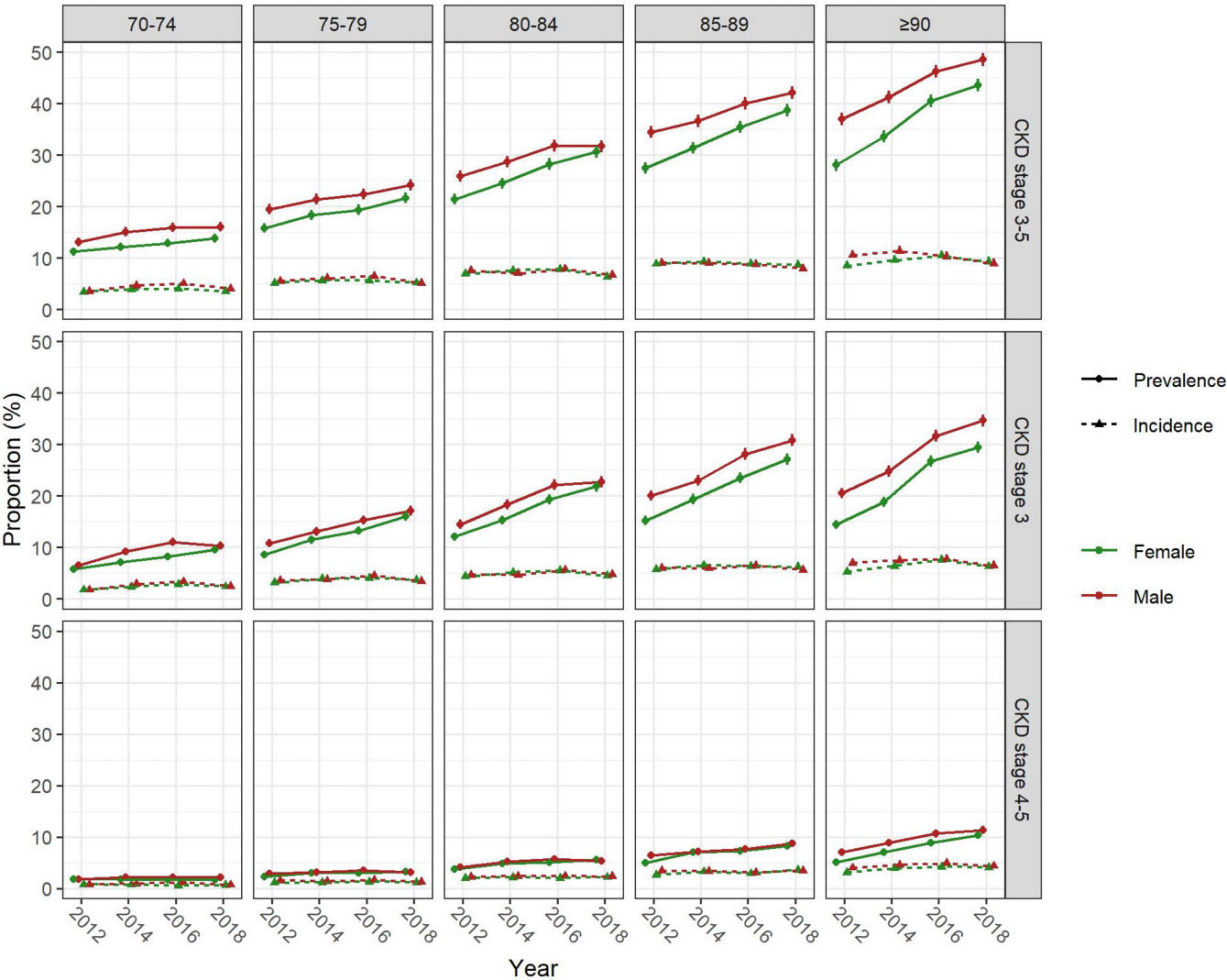
Prevalence and incidence of CKD stratified by sex (color) and age (columns) for overall CKD and CKD stages 3 and 4–5 (rows). Vertical lines represent 95% CIs. Values are interpolated between consecutive years and point estimates dodged around the *x*-axis for graphical display.

**Figure 2:**
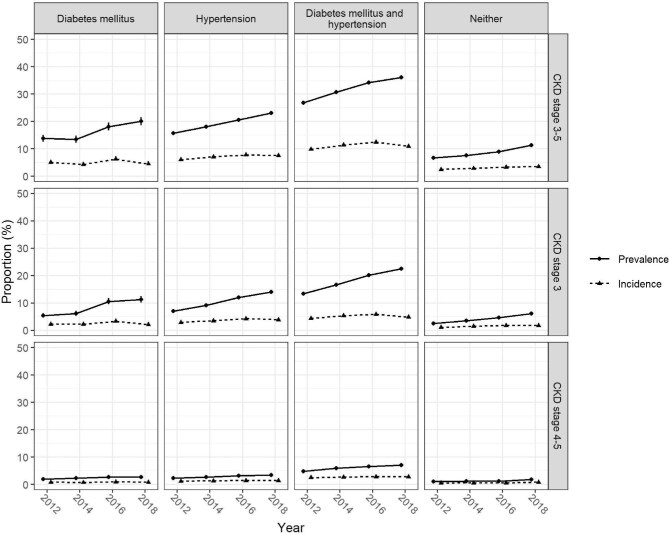
Prevalence and incidence of CKD stratified by comorbidities (columns) for overall CKD and CKD stages 3 and 4–5 (rows). Vertical lines represent 95% CIs. Values are interpolated between consecutive years and point estimates dodged around the *x*-axis for graphical display.

The trends in CKD prevalence and incidence from 2012 to 2018 persisted across stratifications by age, sex and comorbidities, with both estimates rising more steeply in older age groups and in CKD stage 3 compared with stage 4–5 (Fig. 1). Prevalence and incidence of CKD stages 3 and 4–5 were higher in older age, in males compared with females (Fig. 1), and in persons with diabetes or arterial hypertension compared with those without, while co-prevalence of both resulted in the highest estimates (Fig. 2).

When stratified by comorbidities, the results for other QIs revealed partially diverging trends and differences between groups (Supplementary data, Fig. S2), i.e. ACR and dipstick testing were more frequently performed in patients with both prevalent diabetes and arterial hypertension (range: 13.5%–18.1% for QI_ACR_, 57.9 to 60.8% for QI_Dipstick_) compared with patients without these conditions (range: 4.3%–9.2% and 37.2%–49.9%, respectively), while NSAIDs were prescribed less often to patients with both comorbidities (range: 1.5%–4.2% compared with 4.1%–12.8%). In contrast, dual prescriptions of ACE inhibitors and ARBs were more common among patients with both comorbidities (range: 1.5%–5.4%) compared with those without (range: 0.0%–0.5%).

Stratifications by age and sex showed results largely consistent with the main analysis. However, ACR and dipstick testing were performed less frequently with increasing age, and dipstick testing was less common in women compared with men aged ≥80 years (Supplementary data, Table S7, Fig. S3). No considerable differences were observed when analyses were stratified by region of residence (Supplementary data, Fig. S4).

Stratification by healthcare service providers revealed notable differences in CKD prevalence and incidence (Supplementary  data, Table S8). Among patients seen by GPs only, CKD stages 3–5 prevalence was 7.1%, compared with 4.4% for kidney specialists, and 3.3% or lower for other groups in 2012. Prevalence of advanced CKD (stages 4–5) was highest among patients seen by kidney specialists and those hospitalized. Notably, CKD stage 3 prevalence in patients seen by GPs only more than tripled from 2.1% in 2012 to 6.8% in 2018. CKD incidence was highest among hospitalized patients across all stages (Supplementary data, Fig. S5). Stratification by healthcare provider also revealed differences in the performance of quality indicators (Supplementary data, Fig. S6). NSAID prescriptions were similarly low among CKD patients managed by kidney specialists (3.6%–1.7%) and GPs only (3.1%–1.4%), with both showing a declining trend. ACR and dipstick testing were most common in incident CKD patients seen by kidney specialists, with ACR testing rates ranging from 40.7% to 58.0% and dipstick testing from 89.7% to 92.3%.

## DISCUSSION

In this study, we applied new QIs for outpatient healthcare quality in four random cross-sectional samples of claims data and found an increase in coded CKD prevalence in older adults from 2012 to 2018 in Germany. For patients with CKD, non-recommended drug prescriptions such as NSAIDs and dual use of renin–angiotensin system blockers declined over time while ACR and dipstick testing were done less frequently than recommended and remained low.

The CKD prevalence estimates of 17.8% in 2012 and 25.7% in 2018 for our older population are comparable to findings in a large systematic review that included data from America, Europe, Asia and Australia, where CKD prevalence varied from 23% to 36% in individuals aged 64 years and older [10]. However, the Berlin Initiative Study (BIS), a community-dwelling cohort with demographic characteristics similar to those in the present study, reported a CKD prevalence of 37.9% at baseline in 2010 to 2011 [23]. This suggests that our CKD prevalence estimates based on claims data likely underestimate the true prevalence and instead reflect the number of diagnosed and coded cases [23]. This is supported by a recent study investigating referral of CKD patients to nephrologists showing substantial underdiagnosis of CKD in German claims data [11]—an issue that is also recognized in other high-income countries [24]. Additionally, previous claims data analyses have shown limited diagnostic sensitivity for identifying CKD prevalence in Germany [20]. Misclassification and the use of stage-unspecific diagnosis codes likely contribute to an underestimation of CKD prevalence—particularly for stage 3—and should therefore be interpreted with caution. However, claims data remain a key resource for healthcare system research in Germany, which lacks a national CKD registry and the infrastructure to comprehensively link claims with laboratory data as in countries with established electronic patient records [25]. Moreover, the analysis of time trends remains valuable, as it reflects potential changes in diagnostic practices for identifying patients with CKD who may then be eligible for guideline-based treatments.

The rise in CKD prevalence from 2012 to 2018 likely reflects improvements in CKD diagnostics and coding, as well as demographic ageing, which contributes to more individuals with eGFR <60 mL/min/1.73 m² due to the constant decline in kidney function with increasing age [26]. However, CKD prevalence increased across all 5-year age groups between 2012 and 2018, suggesting that ageing alone cannot explain this trend, also considering evidence suggesting stable age-adjusted CKD prevalence [27]. Improved CKD diagnosis, coding and awareness likely contributed to the rise in CKD prevalence. This is supported by a steady increase of CKD prevalence estimates in patients seen by primary care physicians over time and a decrease in stage-unspecified CKD diagnoses, indicating a better adherence to guideline recommendations when diagnosing CKD. Notably, about a quarter of CKD patients still lacked accurate stage diagnosing, indicating further need to improve stage-specific diagnosing. The introduction of disease-management programs by German healthcare funds in 2003, although not specific to CKD, may have indirectly helped by improving management of diabetes and heart failure, leading to earlier CKD detection. These programs were designed to improve the quality of care for chronically ill patients, reduce symptoms associated with chronic diseases, and slow down progression of diabetes and heart failure [28]. While no causal conclusions can be drawn, the implementation of the KDIGO guidelines in 2012 [13] and their adoption into primary care may have impacted CKD coding practices, with digitalization and coding discussions also playing a role.

We also found a decrease in prescriptions of non-recommended drugs, such as NSAIDs, in CKD stage 4–5, along with a reduction in the dual use of renin–angiotensin system blockers (ACE inhibitors and ARBs) and no difference in NSAID prescriptions among patients seen by kidney specialists or GPs. While the frequencies of non-recommended prescriptions of below 5% were lower than those reported in other studies [29–32], both declined by about half between 2012 and 2018. Notably, in exploratory analyses, we also observed an eight percentage points increase in metamizole prescriptions for patients with CKD stages 4–5, suggesting a shift to this kidney-safe pain medication as an alternative to NSAIDs. The reduction in non-recommended prescriptions provides further evidence that awareness and adherence to guidelines such as the KDIGO guidelines is most likely also a contributing factor for improved outpatient care for older patients with CKD.

In contrast, ACR and dipstick testing in patients with incident CKD were performed less frequently than recommended outside kidney specialist care. The proportion of incident CKD patients, particularly those at high risk for CKD progression due to prevalent diabetes and arterial hypertension, who did not receive ACR or dipstick diagnostics was alarmingly high—up to 86.5% and 42.1%, respectively—given the importance of ACR and albuminuria in treatment decisions [6, 33, 34]. The proportion of patients assessed for urine ACR was either lower [31] or comparable to that in other studies [35], with no clear trends observed over time. These stagnating low rates align with findings from the InspeCKD study, which also highlighted inadequate CKD screening in patients at high-risk for CKD development or progression in German GP practices, emphasizing the need for increased awareness among GPs [36]. The National Institute for Health and Care Excellence (NICE) guidelines recommend the use of the Kidney Failure Risk Equation (KFRE) to improve early detection of patients most at risk of kidney failure, but this relies on albuminuria testing, which remains inconsistently performed [37]. The stagnation of ACR testing over time, particularly in the older age groups, may partly reflect an individualized approach to CKD care, where treatment decisions are tailored to prioritize those most likely to benefit from pharmacological interventions.

The quality gaps in healthcare services for older patients with CKD must be addressed with urgency. Our findings may assist healthcare insurers and policymakers in introducing incentives to promote more systematic care and in establishing structured monitoring for patients with CKD. One promising strategy to improve guideline adherence is the expanded use of clinical decision support systems for CKD screening and monitoring [38–40]. This is particularly important given the growing emphasis on early CKD detection and the availability of new drugs—such as sodium-glucose cotransporter 2 (SGLT2) inhibitors and mineralocorticoid receptor antagonists—which have strong cardio- and renoprotective effects and are now included in the updated KDIGO guidelines [41].

### Strengths and limitations

We used a large set of health claims data with random samples based on an *a priori*–defined sample size calculation and study design, which allows the cross-sectional comparison of CKD diagnosis and treatment quality indicators over several years after the KDIGO guidelines introduction in 2012.

Since our data come from a single healthcare fund covering individuals aged ≥70 years in Northeast-Germany, the findings may not necessarily be generalizable to other populations or regions. Furthermore, our analyses are primarily descriptive, limiting causal conclusions for the observed trends. The cross-sectional nature of our data and the absence of patient charts and laboratory values restrict us to diagnosed and coded rather than clinical prevalence and incidence. We present results stratified by kidney specialists, GPs and inpatient services; however, due to the lack of an unambiguous linkage between coded diagnoses, healthcare services and medical specialties in the data limits the interpretability of these findings. Another limitation is the lack of information on in-hospital services—such as albuminuria or dipstick testing—relevant to the quality indicators analyzed. Additionally, our QI definition included albuminuria but not proteinuria testing, which may partly explain the low testing frequency observed. While advanced CKD care and alternatives to dialysis treatment—such as conservative kidney management—are especially relevant for older patients, accurately evaluating these aspects would require integration of patient chart data with claims data, which is currently not feasible. Although this constrains the scope of our recommendations for health policies, in the absence of a kidney registry or electronic patient records in Germany, claims data remain the best data source currently available.

Our results highlight key aspects of healthcare quality for patients with CKD, potentially identifying research gaps and areas for improving guideline implementation. They are consistent with results from other international studies, which have highlighted significant and, in some cases, unacceptably large variations in the care provided to CKD patients, particularly in the monitoring of albuminuria [42–45]. The application of CKD-specific QI measures to healthcare data in a systematic manner is both feasible and can lead to more manageable and effective monitoring of healthcare services. Once specific deficiencies are identified, interventions aimed at improving service delivery should ideally be preceded by cluster randomized controlled trials to evaluate their effectiveness. Further improving diagnostic procedures and stage-specific coding in clinical practice would benefit both clinical outcomes, such as better disease awareness and treatment decisions, as well as the usability of claims data for epidemiologic and healthcare research. Additionally, our findings emphasize the need for electronic database linkage and public accessibility to assess quality of care and support resource allocation in healthcare systems.

### Conclusion

In this study, we analyzed cross-sectional health claims data from biennial random samples between 2012 and 2018 to assess trends in the healthcare service quality for outpatient CKD care after the publication of the KDIGO guidelines. The increasing CKD prevalence estimates, along with declining non-recommended drug prescriptions, suggest improved quality of care. However, the still very low proportions of patients receiving ACR assessment among persons at high-risk for CKD or CKD progression outside of kidney specialist care highlights significant areas for improvement. Future studies modelling long-term trajectories and utilizing nationally linked electronic healthcare data could offer a more comprehensive understanding of healthcare quality and health outcomes for older patients with CKD.

## Supplementary Material

sfaf180_Supplemental_File

## Data Availability

The claims data supporting the findings of this study are not publicly available due to German regulations on sensitive health data and data protection (Bundesdatenschutzgesetz). However, further information, including anonymized and aggregated data, and coding details not subject to data protection regulations, are available from the corresponding author upon reasonable request.
