# Supplementary material for: Chronic kidney disease in older adults: trends in prevalence and healthcare service quality from 2012 to 2018
Source: Clin Kidney J. 2025 Jun 14;18(7):sfaf180. doi: 10.1093/ckj/sfaf180 (PMC12223366; doi:10.1093/ckj/sfaf180)
Supplement: sfaf180_Supplemental_File [file sfaf180_supplemental_file.docx]

# **Supplementary material**

for

**Chronic Kidney Disease in Older Adults: Trends in Prevalence and Healthcare Service Quality from 2012 to 2018**

**List of contents**

Table S1 – Operationalizations and used computational criteria for the numerators and denominators (persons at risk) for all respective quality indicators (QI) within health claims data. 2

Table S2 – Year-, sex, and age group-specific weights used for the standardization procedure. 5

Table S3 – Operationalizations and used computational criteria for sociodemographic and clinical variables within health claims data. 6

Table S4 – Operationalizations and used computational criteria for the numerators and denominators (persons at risk) for all respective quality indicators (QI) within health claims data for stratifications by healthcare service provider. 7

Table S5 – Prevalence and incidence of CKD stage 3 and stage 4‑5. 9

Table S6 – Prevalence of stage-specified and stage-unspecified CKD. 10

Figure S1 – Prevalence of stage-specified and stage-unspecified CKD, stratified by sex and age. 11

Figure S2 – Quality indicators (QI) for the outpatient healthcare service quality for patients with CKD, stratified by comorbidities. 12

Table S7 – Prevalence, incidence, and quality indicators (QI) for the outpatient healthcare service quality for patients with CKD with trend-tests over time, stratified by sex and age. 13

Figure S3 – Quality indicators (QI) for the outpatient healthcare service quality for patients with CKD, stratified by sex and age. 17

Figure S4 – Prevalence, incidence, and quality indicators (QI) for the outpatient healthcare service quality for patients with CKD, stratified by region of residence. 18

Table S8 – Prevalence, incidence, and quality indicators (QI) for the outpatient healthcare service quality for patients with CKD, stratified by healthcare service provider. 19

Figure S5 – Prevalence and incidence of CKD, stratified by healthcare service provider. 21

Figure S6 – Quality indicators (QI) for the outpatient healthcare service quality for patients with CKD, stratified by healthcare service provider. 22

Table S9 – STROBE checklist 23

Table S1 – Operationalizations and used computational criteria for the numerators and denominators (persons at risk) for all respective quality indicators (QI) within health claims data.

| **QI** | **Description** | **Numerator** | **Denominator (*persons at risk*)** |
| --- | --- | --- | --- |
| **Prevalence** | **Prevalent CKD:**  Proportion of patients ≥70 years with diagnosed CKD | 1. At least one ICD-10-GM code N18.3, N18.4, N18.5, N18.8x, N18.9, or N19^a^ within one year | All persons within the respective year tranche |
|  | **Prevalent CKD stage 3:**  Proportion of patients ≥70 years with diagnosed CKD stage 3 | 1. At least one ICD-10-GM code N18.3^a^ within one year   *AND*   1. No ICD‑10‑GM code N18.4, N18.5^a^ within the same year | All persons within the respective year tranche |
|  | **Prevalent CKD stage 4‑5:**  Proportion of patients ≥70 years with diagnosed CKD stage 4‑5 | 1. At least one ICD-10-GM code N18.4 or N18.5^a^ within one year | All persons within the respective year tranche |
| **Incidence** | **Incident CKD:**  Proportion of patients ≥70 years with newly diagnosed CKD | 1. Prevalent CKD   *AND*   1. No ICD-10-GM code N18.3, N18.4, N18.5, N18.8x, N18.9, or N19^a^ within one year preceding the respective year tranche | All persons without ICD-10-GM codes N18.3, N18.4, N18.5, N18.8x, N18.9, or N19^a^ within the year preceding the respective year tranche |
|  | **Incident CKD stage 3:**  Proportion of patients ≥70 years with newly diagnosed CKD stage 3 | 1. Prevalent CKD stage 3   *AND*   1. No ICD-10-GM code N18.3, N18.4, or N18.5^a^ within one year preceding the respective year tranche | All persons without ICD-10-GM codes N18.3, N18.4, or N18.5^a^ within the year preceding the respective year tranche |
|  | **Incident CKD stage 4‑5:**  Proportion of patients ≥70 years with newly diagnosed CKD stage 4‑5 | 1. Prevalent CKD stage 4‑5   *AND*   1. No ICD-10-GM code N18.4, or N18.5^a^ within one year preceding the respective year tranche | All persons without ICD-10-GM codes N18.4, or N18.5^a^ within the year preceding the respective year tranche |
|  |  |  |  |
| **QI_ACR_**  ***Table S1*** *– continued* | Proportion of patients ≥70 years with incident CKD who had a urinary albumin/creatinine ratio (ACR) determined | 1. Incident CKD *AND* 2. Urinary albumin/creatinine ratio (ACR) determined at least once within the quarter of incidence admission: GOP code 32435 (albumin) was billed concurrently with either 32066 or 32067 (creatinine) | All persons with incident CKD |
| **QI_Dipstick_** | Proportion of patients ≥70 years with incident CKD who had a dipstick-test for protein, glucose, erythrocytes, leukocytes, and nitrite | 1. Incident CKD *AND* 2. Dipstick-test for protein, glucose, erythrocytes, leukocytes, and nitrite (GOP codes 32030, 32880) in the same quarter as the diagnosis | All persons with incident CKD |
| **QI_ACEi+ARB_** | Proportion of patients ≥70 years with CKD who had multiple (≥2) dual prescriptions of angiotensin-converting-enzyme (ACE) inhibitors and angiotensin II receptor blockers (ARBs) over at least 2 quarters within 3 quarters after the quarter with the first simultaneous prescription (non-recommended) | 1. Prevalent CKD   *AND*   1. A dual prescription of ACE inhibitors (ACEi) and ARBs in the same year quarter as the prevalence diagnosis: at least one drug prescription with ATC code C09A, C09B, C09BB, or C09BX (ACEi) and one drug prescription with ATC code C09C or C09D (ARB) within the same year quarter   *AND*   1. At least one other quarter with a dual prescription within 3 quarters after the quarter with the first dual prescription   *UNLESS*   - 1. the first concomitant prescription was in Q3 and no second concomitant prescription was seen in Q4 of that year or Q1 of the following year   *OR*   - 1. the first dual prescription was in Q4 and no second dual prescription was seen in Q1 of the following year | All persons with prevalent CKD  *UNLESS*   - 1. their first dual prescription was in Q3 and no second dual prescription was seen in Q4 of that year or Q1 of the following year   *OR*   - 1. their first dual prescription was in Q4 and no second dual prescription was seen in Q1 of the following year |
| **QI_NSAID_** | Proportion of patients ≥70 years with CKD stage 4-5 (eGFR <30 ml/min/1.73m²) for whom nonsteroidal anti-inflammatory drugs (NSAIDs) were prescribed (*non-recommended*) | 1. Prevalent CKD stage 4‑5   *AND*   1. At least one ICD-10-GM code N18.4 or N18.5^a^ within one year preceding the respective year tranche *AND* 2. At least one drug prescription with ATC code M01A or N02BA01 (excluding B01AC06 and M01AX) within the quarter of CKD stage 4-5 prevalence diagnosis or the preceding three quarters | All persons with prevalent CKD stage 4‑5  *AND*  At least one ICD-10-GM code N18.4 or N18.5^a^ within one year preceding the respective year tranche |
| **Metamizole**  ***Table S1*** *– continued* | Proportion of patients ≥70 years with CKD stage 4-5 (eGFR <30 ml/min/1.73m²) for whom Metamizole was prescribed | 1. Prevalent CKD stage 4‑5 *AND* 2. At least one ICD-10-GM code N18.4 or N18.5^a^ within one year preceding the respective year tranche   *AND*   1. At least one drug prescription with ATC code N02BB02 or R05XA07 within the quarter of CKD stage 4-5 prevalence diagnosis or the preceding three quarters | All persons with prevalent CKD stage 4‑5  *AND*  At least one ICD-10-GM code N18.4 or N18.5^a^ within one year preceding the respective year tranche |
| *Abbreviations:* CKD: Chronic kidney disease; ICD‑10‑GM: Diagnoses in accordance with the International Statistical Classification of Diseases and Related Health Problems, 10^th^ Revision, German Modification; GOP: Codes of the *Uniform Value Scale* or *Doctors’ fee schedule* for single outpatient treatments or services (*Gebührenordnungsposition*); ATC: anatomical therapeutic chemical code for drugs. *Notes:* All criteria were applied to all single year tranches independently from one another (2012, 2014, 2016, and 2018 each). We used only outpatient diagnoses with the diagnosis type “secure” or inpatient “main” or “secondary” discharge diagnoses.  ^a^ Older ICD-9-GM Version codes (before 2010) for CKD were recoded to their ICD-10-GM equivalent as follows: N18.81 to N18.1, N18.82 to N18.2, N18.83 to N18.3, N18.84 to N18.4, N18.0 to N18.5. | | | |

Table S2 – Year-, sex, and age group-specific weights used for the standardization procedure.

| **Sex** | **Age group** | **2012** | **2014** | **2016** | **2018** |
| --- | --- | --- | --- | --- | --- |
| Males | 70-74 | 0.1851 | 0.1676 | 0.1321 | 0.1195 |
|  | 75-79 | 0.1252 | 0.1375 | 0.1521 | 0.1476 |
|  | 80-84 | 0.0647 | 0.0694 | 0.0838 | 0.0984 |
|  | 85-89 | 0.0242 | 0.0299 | 0.0356 | 0.0379 |
|  | ≥90 | 0.0081 | 0.0086 | 0.0106 | 0.0131 |
| Females | 70-74 | 0.2195 | 0.1961 | 0.1543 | 0.1391 |
|  | 75-79 | 0.1677 | 0.1813 | 0.1967 | 0.1882 |
|  | 80-84 | 0.1058 | 0.1077 | 0.1258 | 0.1451 |
|  | 85-89 | 0.0646 | 0.0664 | 0.0699 | 0.0702 |
|  | ≥90 | 0.0351 | 0.0357 | 0.0391 | 0.0408 |
| *Notes:* We used demographic data from the Federal Statistical Office in Germany (https://www-genesis.destatis.de/datenbank/beta/statistic/12411/table/12411-0013/) for the states from which the study samples were drawn (Berlin, Brandenburg, and Mecklenburg-Vorpommern) to estimate the weights. | | | | | |

Table S3 – Operationalizations and used computational criteria for sociodemographic and clinical variables within health claims data.

| **Variable** | **Description** | **Computational criteria** |
| --- | --- | --- |
| Chronic dialysis | Exclusion of patients with chronic dialysis to refer to non-dialysis dependent CKD | Within one year, either   1. Outpatient dialysis:    1. ICD‑10‑GM Z49.1–2 *AND*    2. ICD‑10‑GM Z99.2 *AND*    3. GOP 13610 or 13611   *OR*   1. Day-care dialysis (only diagnoses or OPS-codes billed as day-care):    1. ICD‑10‑GM Z49.1–2 *AND*    2. OPS 8‑853, 8‑854, 8‑855, or 8‑857 |
| *Comorbidities*  Diabetes mellitus | Persons with diagnosed or treated diabetes mellitus | Within one year, either   1. At least one inpatient ICD‑10‑GM code E10–14 as “main” discharge diagnosis   *OR*   1. At least one outpatient ICD‑10‑GM code E10–14 with diagnosis type “secure” or “condition after” in at least two year quarters   *OR*   1. At least one outpatient ICD‑10‑GM code E10–14 with diagnosis type “secure” or “condition after”   *AND*  At least one dispensed antidiabetic drug (ATC‑code A10A or A10B) |
| Arterial hypertension | Persons with diagnosed and treated arterial hypertension | 1. At least one ICD‑10‑GM code I10–13 or I15   *AND*   1. At least one dispensed anti-hypertensive drug (ATC‑code C02, C03A, C03B, C03D, C03EA, C03EB01, C03EB21, C03EC, C03ED01, C07, C08, C09, C10BX03, or C10BX04, excluding C02KP) |
| *Abbreviations:* ICD‑10‑GM: Diagnoses in accordance with the International Statistical Classification of Diseases and Related Health Problems, 10^th^ Revision, German Modification; GOP: Codes of the *Uniform Value Scale* or *Doctors’ fee schedule* for single outpatient treatments or services (*Gebührenordnungsposition*); OPS: Operational and procedural codes for inpatient treatments and services; ATC: anatomical therapeutic chemical codes for drugs. *Notes:* All criteria were applied to all single year tranches independently from one another (2012, 2014, 2016, and 2018 each). If not stated otherwise, we used only outpatient diagnoses with the diagnosis type “secure” or inpatient “main” or “secondary” discharge diagnoses. | | |

Table S4 – Operationalizations and used computational criteria for the numerators and denominators (persons at risk) for all respective quality indicators (QI) within health claims data for stratifications by healthcare service provider.

|  | **Kidney specialists** | **Hospital^a^ + GP** | **GP only** | **Hospital^a^** | **Other** |
| --- | --- | --- | --- | --- | --- |
| **Definition** | Patients with a CKD diagnosis billed by a nephrologist or urologist | Patients with a CKD diagnosis billed by a GP and a hospital *AND* not by kidney specialists | Patients with a CKD diagnosis by a GP *AND* not by a kidney specialists or hospital | Patients with a CKD diagnosis billed by a hospital *AND* not by a kidney specialist or GP | Patients with a CKD diagnosis by other outpatient practitioners *AND* not by a kidney specialist, GP, or hospital |
| **Used reference codes** | Outpatient medical specialty group codes: 29, 67, identified via case numbers | Inpatient diagnoses (all) and outpatient medical specialty group codes: 1, 2, 3, identified via case numbers | Outpatient medical specialty group codes: 1, 2, 3, identified via case numbers | Inpatient diagnoses (all) | Outpatient medical specialty group codes: all others but 1, 2, 3, 29, 67, identified via case numbers |
| **Quality indicators** |  |  |  |  |  |
| **Prevalence** – CKD stage 3-5 | | | | | |
| Numerator: | 1. At least one outpatient ICD-10-GM code N18.3, N18.4, N18.5, N18.8x, N18.9, or N19^b^ within one year by | | | | |
|  | 2.1 a kidney specialist | 2.2 a hospital *AND* a GP | 2.3 a GP | 2.4 a hospital | 2.5 others |
| Denominator: | All persons within the respective year tranche | | | | |
| **Prevalence** – CKD stage 3 | | | | | |
| Numerator: | 1. At least one outpatient ICD-10-GM code N18.3^b^ within one year by | | | | |
|  | 2.1 a kidney specialist | 2.2 a hospital *AND* a GP | 2.3 a GP | 2.4 a hospital | 2.5 others |
| Denominator: | All persons within the respective year tranche | | | | |
| **Prevalence** – CKD stage 4-5 | | | | | |
| Numerator: | 1. At least one outpatient ICD-10-GM code N18.4 or N18.5^b^ within one year by | | | | |
|  | 2.1 a kidney specialist | 2.2 a hospital *AND* a GP | 2.3 a GP | 2.4 a hospital | 2.5 others |
| Denominator: | All persons within the respective year tranche | | | | |
| **Incidence** – CKD stage 3-5 | | | | | |
| Numerator: | 1. At least one outpatient ICD-10-GM code N18.3, N18.4, N18.5, N18.8x, N18.9, or N19^b^ within one year by | | | | |
|  | 2.1 a kidney specialist | 2.2 a hospital *AND* a GP | 2.3 a GP | 2.4 a hospital | 2.5 others |
|  | 3. No ICD-10-GM code N18.3, N18.4, N18.5, N18.8x, N18.9, or N19^b^ within one year preceding the respective year tranche | | | | |
| Denominator: | All persons without ICD-10-GM codes N18.3, N18.4, N18.5, N18.8x, N18.9, or N19^b^ within the year preceding the respective year tranche | | | | |
| **Incidence** – CKD stage 3  ***Table S4*** *– continued* | | | | | |
| Numerator: | 1. At least one outpatient ICD-10-GM code N18.3^b^ within one year by | | | | |
|  | 2.1 a kidney specialist | 2.2 a hospital *AND* a GP | 2.3 a GP | 2.4 a hospital | 2.5 others |
|  | 3. No ICD-10-GM code N18.3, N18.4, or N18.5^b^ within one year preceding the respective year tranche | | | | |
| Denominator: | All persons without ICD-10-GM codes N18.3, N18.4, or N18.5^b^ within the year preceding the respective year tranche | | | | |
| **Incidence** – CKD stage 4-5 | | | | | |
| Numerator: | 1. At least one outpatient ICD-10-GM code N18.4 or N18.5^b^ within one year by | | | | |
|  | 2.1 a kidney specialist | 2.2 a hospital *AND* a GP | 2.3 a GP | 2.4 a hospital | 2.5 others |
|  | 3. No ICD-10-GM code N18.4 or N18.5^b^ within one year preceding the respective year tranche | | | | |
| Denominator: | All persons without ICD-10-GM codes N18.4 or N18.5^b^ within the year preceding the respective year tranche | | | | |
| **QI_ACR_, QI_Dipstick_, QI_ACEi+ARB_, QI_NSAID_, Metamizole** | | | | | |
| Numerator: | Definition as in the overall analysis (see Table S1) | | | | |
| Denoninator: | All persons with prevalent or incident CKD (as defined above; regarding stage selection, see Table S1) by | | | | |
|  | 2.1 a kidney specialist | 2.2 a hospital AND a GP | 2.3 a GP | 2.4 a hospital | 2.5 others |
| Abbreviations: CKD: Chronic kidney disease; ICD‑10‑GM: Diagnoses in accordance with the International Statistical Classification of Diseases and Related Health Problems, 10^th^ Revision, German Modification; GP: General practitioner. Notes: All criteria were applied to all single year tranches independently from one another (2012, 2014, 2016, and 2018 each). We used only outpatient diagnoses with the diagnosis type “secure” or inpatient “main” or “secondary” discharge diagnoses.  ^a^ Patients in this group represent those who received their CKD diagnosis (either prevalence or incidence) during hospitalization. Single procedures, such as ACR or dipstick tests, performed in-hospital cannot be identified in claims data. To account for potential variations in subsequent outpatient care (e.g., after referral), we differentiated in this sensitivity analysis between those who received a CKD diagnosis from a general practitioner (GP) within the same year (hospital + GP) and those who did not (hospital). ^b^ Older ICD-9-GM Version codes (before 2010) for CKD were recoded to their ICD-10-GM equivalent as follows: N18.81 to N18.1, N18.82 to N18.2, N18.83 to N18.3, N18.84 to N18.4, N18.0 to N18.5. | | | | | |

Table S5 – Prevalence and incidence of CKD stage 3 and stage 4‑5.

|  | **2012** | | **2014** | | **2016** | | **2018** | | Δ |
| --- | --- | --- | --- | --- | --- | --- | --- | --- | --- |
| ***Prevalence of CKD stage 3:*** Proportion of patients ≥70 years with diagnosed CKD stage 3 | | | | | | | | | |
| Persons at risk^a^, *n* | 61,970 |  | 61,986 |  | 62,018 |  | 61,994 |  |  |
| Proportion^b^, *% (95%‑CI)* | 8.2 | (8.0; 8.5) | 10.7 | (10.4; 10.9) | 13.7 | (13.4; 13.9) | 15.7 | (15.4; 16.0) | +7.5 |
| ***Prevalence of CKD stage 4‑5:*** Proportion of patients ≥70 years with diagnosed CKD stage 4‑5 | | | | | | | | | |
| Persons at risk^a^, *n* | 61,970 |  | 61,986 |  | 62,018 |  | 61,994 |  |  |
| Proportion^b^, *% (95%‑CI)* | 2.8 | (2.7; 3.0) | 3.6 | (3.4; 3.7) | 4.0 | (3.9; 4.2) | 4.4 | (4.2; 4.6) | +1.6 |
| ***Incidence of CKD stage 3:*** Proportion of patients ≥70 years with newly diagnosed CKD stage 3 | | | | | | | | | |
| Persons at risk^a^, *n (%)* | 56,093 | (90.5) | 54,095 | (87.3) | 52,371 | (84.4) | 49,996 | (80.6) |  |
| Proportion^b^, *% (95%‑CI)* | 3.3 | (3.1; 3.4) | 4.1 | (4.0; 4.3) | 5.0 | (4.8; 5.2) | 4.6 | (4.4; 4.7) | +1.3 |
| ***Incidence of CKD stage 4‑5:*** Proportion of patients ≥70 years with newly diagnosed CKD stage 4‑5 | | | | | | | | | |
| Persons at risk^a^, *n (%)* | 60,325 | (97.3) | 59,820 | (96.4) | 59,517 | (96.0) | 59,214 | (95.5) |  |
| Proportion^b^, *% (95%‑CI)* | 1.5 | (1.4; 1.6) | 1.7 | (1.6; 1.8) | 1.8 | (1.7; 1.9) | 1.8 | (1.7; 1.9) | +0.3 |
| *Abbreviations:* 95%‑CI: 95% confidence interval. Δ: Difference from 2012 to 2018.   1. The number of persons at risk indicates how many persons were eligible for testing of the respective QI (denominator). E.g., for incidence: only persons who did not have a CKD diagnosis in the respective previous year. 2. The proportions for all QI were standardized using sex-, age group-, and year-specific weights for the population aged ≥70 years in Northeast-Germany. | | | | | | | | | |

Table S6 – Prevalence of stage-specified and stage-unspecified CKD.

|  | **2012** | | **2014** | | **2016** | | **2018** | | Δ |
| --- | --- | --- | --- | --- | --- | --- | --- | --- | --- |
| ***Prevalence of CKD stage 3-5:*** Proportion of patients ≥70 years with diagnosed CKD stage 3-5 (ICD-10-GM N18.3-5, N18.8-9, N19) | | | | | | | | | |
| Persons at risk^a^, *n* | 61,970 |  | 61,986 |  | 62,018 |  | 61,994 |  |  |
| Proportion^b^, *% (95%‑CI)* | 17.8% | (17.5; 18.1) | 20.5% | (20.2; 20.8) | 23.5% | (23.1; 23.8) | 25.7% | (25.4; 26.1) | +8.0 |
| ***CKD stage specified:*** Proportion of patients ≥70 years who received a stage-specific CKD diagnosis (ICD-10-GM N18.3-.5) | | | | | | | | | |
| Proportion^b^, *% (95%‑CI)* | 11.1% | (10.8; 11.3) | 14.2% | (14.0; 14.5) | 17.7% | (17.4; 18.0) | 20.1% | (19.7; 20.4) | +9.0 |
| ***CKD stage unspecified:*** Proportion of patients ≥70 years who received only stage-unspecific CKD diagnoses (ICD-10-GM N18.8-9, N19) | | | | | | | | | |
| Proportion^b^, *% (95%‑CI)* | 6.7% | (6.5; 6.9) | 6.3% | (6.1; 6.5) | 5.8% | (5.6; 6.0) | 5.7% | (5.5; 5.9) | -1.0 |
| *Abbreviations:* 95%‑CI: 95% confidence interval. Δ: Difference from 2012 to 2018.   1. The number of persons at risk indicates how many persons were eligible for testing of the respective QI (denominator). E.g., for incidence: only persons who did not have a CKD diagnosis in the respective previous year. 2. The proportions for all QI were standardized using sex-, age group-, and year-specific weights for the population aged ≥70 years in Northeast-Germany. | | | | | | | | | |


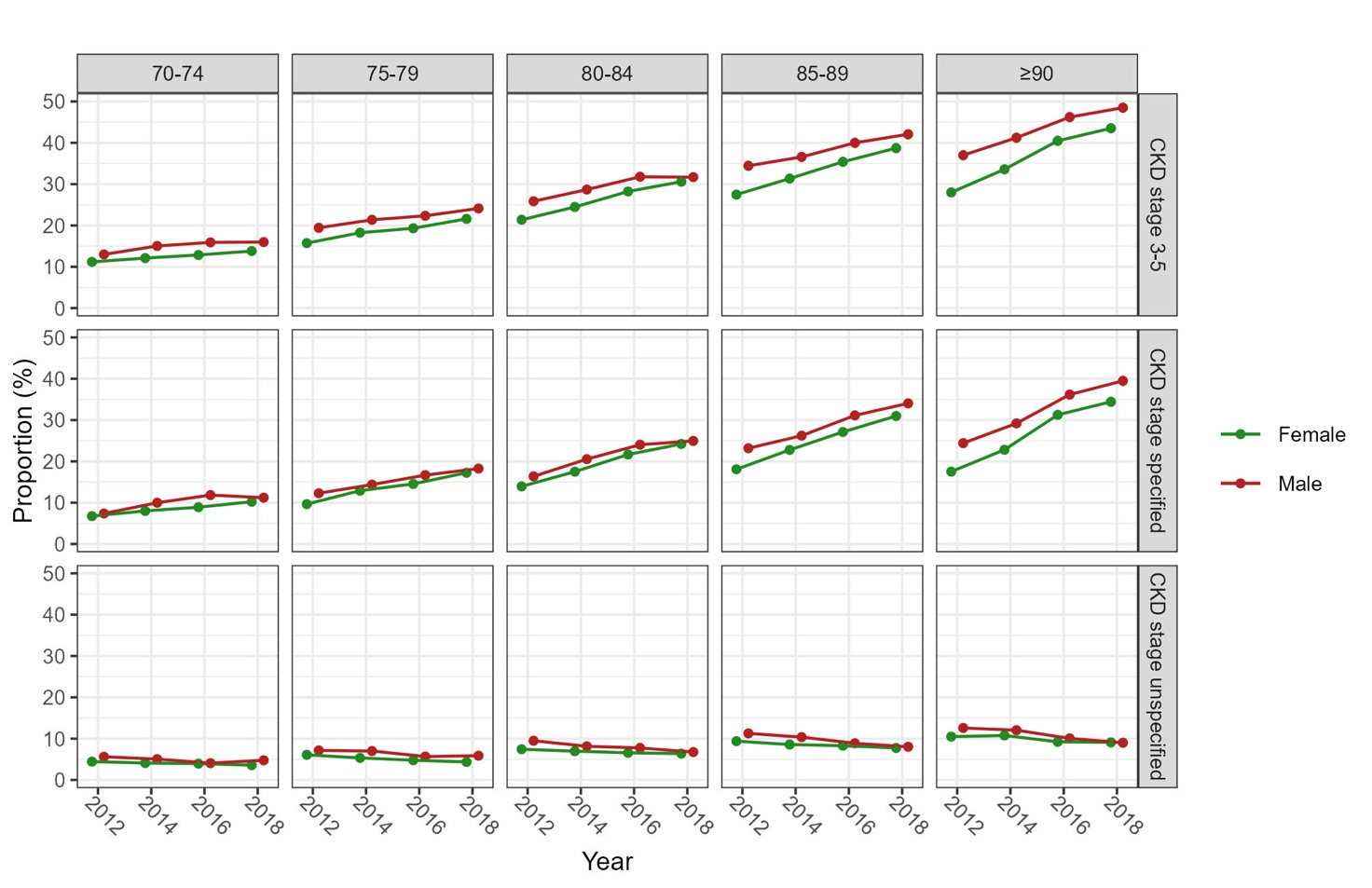


Figure S1 – Prevalence of stage-specified and stage-unspecified CKD, stratified by sex and age. Vertical lines represent 95% confidence intervals. Values are interpolated between consecutive years and point estimates dodged around the x-axis for graphical display.


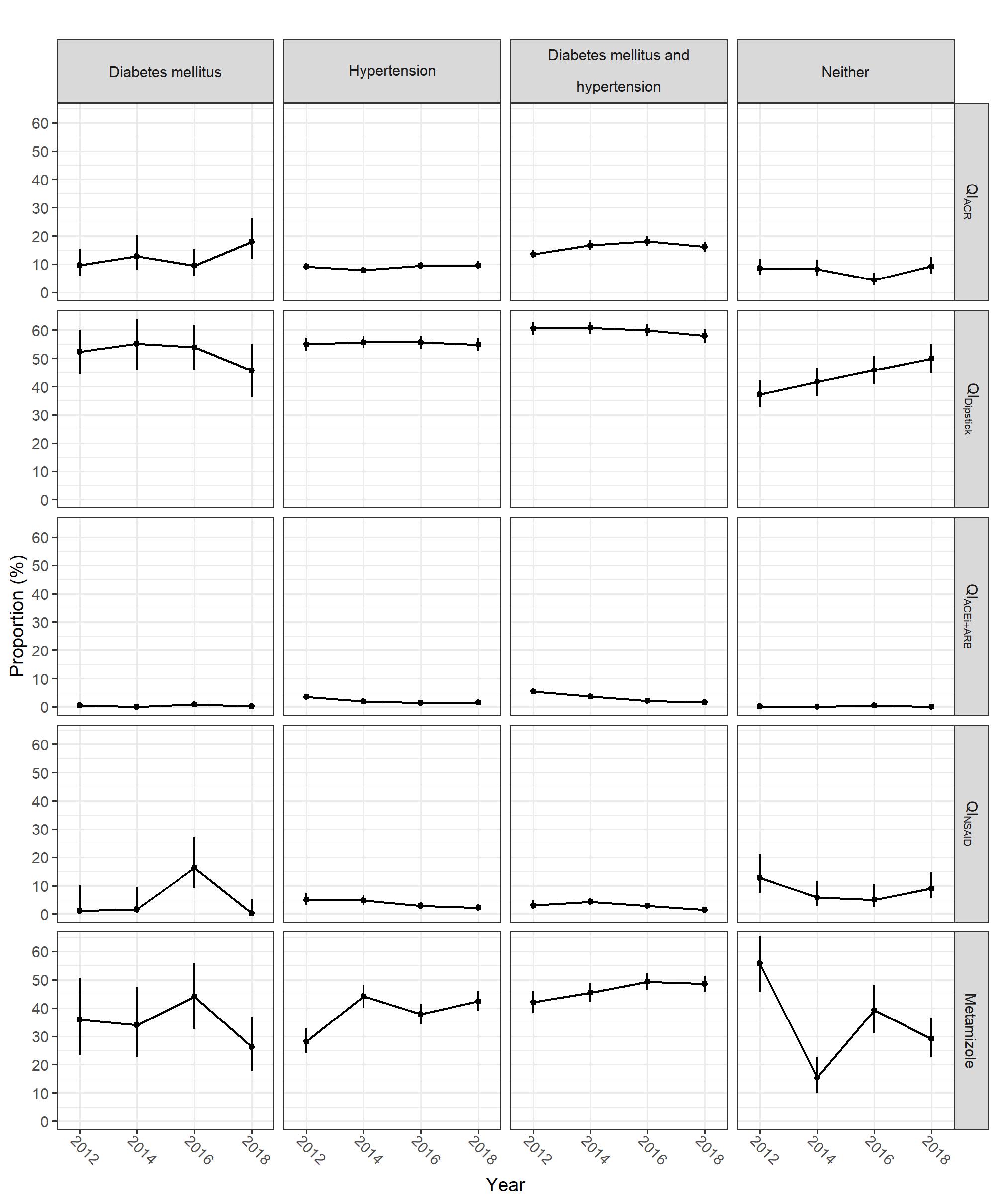


Figure S2 – Quality indicators (QI) for the outpatient healthcare service quality for patients with CKD, stratified by comorbidities. Vertical lines represent 95% confidence intervals. Values are interpolated between consecutive years and point estimates dodged around the x-axis for graphical display.

Table S7 – Prevalence, incidence, and quality indicators (QI) for the outpatient healthcare service quality for patients with CKD with trend-tests over time, stratified by sex and age.

| **Sex** | **Age** |  | **2012** | | |  |  | **2014** | | |  |  | **2016** | | |  |  | **2018** | | |  |  | **Trend^a^** | | |  |  |  |
| --- | --- | --- | --- | --- | --- | --- | --- | --- | --- | --- | --- | --- | --- | --- | --- | --- | --- | --- | --- | --- | --- | --- | --- | --- | --- | --- | --- | --- |
|  |  | At risk | | % | (95%‑CI) | | At risk | | % | (95%‑CI) | | At risk | | % | (95%‑CI) | | At risk | | % | (95%‑CI) | | Δ | | *p* |  | | |  |
| **Prevalence:** Proportion of patients ≥70 years with diagnosed CKD | | | | | | | | | | | | | | | | | | | | | | | | | | | |  |
| Males | 70-74 | 6,199 | | 13.0 | (12.2; 13.9) | | 6,192 | | 15.0 | (14.2; 15.9) | | 6,199 | | 15.9 | (15.0; 16.8) | | 6,194 | | 16.0 | (15.1; 16.9) | | 3.0 | | <0.0001 | * | | |  |
|  | 75-79 | 6,189 | | 19.4 | (18.5; 20.4) | | 6,191 | | 21.4 | (20.4; 22.4) | | 6,195 | | 22.3 | (21.3; 23.4) | | 6,185 | | 24.1 | (23.1; 25.2) | | 4.7 | | <0.0001 | * | | |  |
|  | 80-84 | 6,177 | | 25.9 | (24.8; 27.0) | | 6,188 | | 28.7 | (27.6; 29.8) | | 6,195 | | 31.8 | (30.6; 33.0) | | 6,197 | | 31.7 | (30.5; 32.9) | | 5.8 | | <0.0001 | * | | |  |
|  | 85-89 | 6,187 | | 34.4 | (33.3; 35.6) | | 6,186 | | 36.6 | (35.4; 37.8) | | 6,201 | | 40.0 | (38.8; 41.2) | | 6,187 | | 42.1 | (40.8; 43.3) | | 7.6 | | <0.0001 | * | | |  |
|  | ≥90 | 6,201 | | 37.0 | (35.8; 38.2) | | 6,206 | | 41.2 | (40.0; 42.5) | | 6,205 | | 46.2 | (45.0; 47.5) | | 6,196 | | 48.5 | (47.3; 49.7) | | 11.5 | | <0.0001 | * | | |  |
| Females | 70-74 | 6,205 | | 11.2 | (10.4; 12.0) | | 6,204 | | 12.1 | (11.3; 12.9) | | 6,207 | | 12.9 | (12.0; 13.7) | | 6,206 | | 13.8 | (13.0; 14.7) | | 2.6 | | <0.0001 | * | | |  |
|  | 75-79 | 6,203 | | 15.7 | (14.8; 16.7) | | 6,205 | | 18.2 | (17.3; 19.2) | | 6,200 | | 19.3 | (18.4; 20.3) | | 6,204 | | 21.6 | (20.6; 22.6) | | 5.9 | | <0.0001 | * | | |  |
|  | 80-84 | 6,204 | | 21.4 | (20.4; 22.4) | | 6,203 | | 24.5 | (23.4; 25.6) | | 6,200 | | 28.2 | (27.1; 29.4) | | 6,207 | | 30.6 | (29.5; 31.8) | | 9.2 | | <0.0001 | * | | |  |
|  | 85-89 | 6,194 | | 27.5 | (26.4; 28.6) | | 6,205 | | 31.3 | (30.2; 32.5) | | 6,203 | | 35.4 | (34.2; 36.6) | | 6,204 | | 38.7 | (37.5; 39.9) | | 11.3 | | <0.0001 | * | | |  |
|  | ≥90 | 6,211 | | 28.0 | (26.9; 29.1) | | 6,206 | | 33.6 | (32.4; 34.8) | | 6,213 | | 40.5 | (39.3; 41.7) | | 6,214 | | 43.5 | (42.3; 44.8) | | 15.5 | | <0.0001 | * | | |  |
| **Incidence:** Proportion of patients ≥70 years with newly diagnosed CKD | | | | | | | | | | | | | | | | | | | | | | | | | | | |  |
| Males | 70-74 | 5,494 | | 3.9 | (3.5; 4.5) | | 5,427 | | 5.3 | (4.7; 5.9) | | 5,420 | | 5.8 | (5.2; 6.4) | | 5,318 | | 4.7 | (4.2; 5.3) | | 0.8 | | 0.0321 |  | | |  |
|  | 75-79 | 5,185 | | 6.6 | (5.9; 7.3) | | 5,100 | | 7.3 | (6.6; 8.1) | | 5,078 | | 7.9 | (7.2; 8.7) | | 4,860 | | 6.5 | (5.8; 7.2) | | -0.1 | | 0.7729 |  | | |  |
|  | 80-84 | 4,878 | | 9.6 | (8.8; 10.4) | | 4,673 | | 9.3 | (8.5; 10.1) | | 4,539 | | 10.7 | (9.8; 11.6) | | 4,474 | | 9.3 | (8.5; 10.2) | | -0.3 | | 0.7693 |  | | |  |
|  | 85-89 | 4,408 | | 12.7 | (11.7; 13.7) | | 4,270 | | 13.0 | (12.0; 14.0) | | 4,057 | | 13.3 | (12.3; 14.3) | | 3,846 | | 12.7 | (11.7; 13.8) | | 0.0 | | 0.8373 |  | | |  |
|  | ≥90 | 4,288 | | 15.2 | (14.2; 16.3) | | 4,071 | | 17.3 | (16.2; 18.5) | | 3,720 | | 17.1 | (15.9; 18.3) | | 3,460 | | 15.9 | (14.7; 17.2) | | 0.7 | | 0.3652 |  | | |  |
| Females | 70-74 | 5,643 | | 3.7 | (3.3; 4.3) | | 5,602 | | 4.2 | (3.7; 4.8) | | 5,557 | | 4.5 | (4.0; 5.1) | | 5,462 | | 3.9 | (3.5; 4.5) | | 0.2 | | 0.4575 |  | | |  |
|  | 75-79 | 5,424 | | 5.8 | (5.2; 6.5) | | 5,278 | | 6.6 | (5.9; 7.3) | | 5,222 | | 6.6 | (6.0; 7.3) | | 5,028 | | 6.3 | (5.7; 7.1) | | 0.5 | | 0.2596 |  | | |  |
|  | 80-84 | 5,137 | | 8.3 | (7.6; 9.1) | | 4,958 | | 9.4 | (8.6; 10.3) | | 4,741 | | 10.2 | (9.4; 11.1) | | 4,519 | | 8.7 | (7.9; 9.5) | | 0.3 | | 0.2773 |  | | |  |
|  | 85-89 | 4,835 | | 11.3 | (10.4; 12.2) | | 4,578 | | 12.6 | (11.7; 13.6) | | 4,326 | | 12.6 | (11.7; 13.7) | | 4,122 | | 13.0 | (12.0; 14.0) | | 1.7 | | 0.0163 |  | | |  |
|  | ≥90 | 4,740 | | 11.1 | (10.2; 12.0) | | 4,421 | | 13.4 | (12.4; 14.4) | | 4,071 | | 15.9 | (14.8; 17.0) | | 3,814 | | 15.0 | (14.0; 16.2) | | 4.0 | | <0.0001 | * | | |  |
|  | | | | | | | | | | | | | | | | | | | | | | | | | | | |  |
| **QI_ACR_:** Proportion of patients ≥70 years with incident CKD who had a urinary albumin/creatinine ratio determined  ***Table S7*** *– continued* | | | | | | | | | | | | | | | | | | | | | | | | | | | |  |
| Males | 70-74 | 217 | | 14.3 | (10.3; 19.6) | | 285 | | 16.5 | (12.6; 21.2) | | 312 | | 15.1 | (11.5; 19.5) | | 250 | | 16.0 | (12.0; 21.1) | | 1.7 | | 0.7657 |  | | |  |
|  | 75-79 | 340 | | 10.9 | (8.0; 14.6) | | 373 | | 10.5 | (7.7; 14.0) | | 401 | | 13.2 | (10.2; 16.9) | | 315 | | 13.7 | (10.3; 17.9) | | 2.8 | | 0.1564 |  | | |  |
|  | 80-84 | 467 | | 9.6 | (7.3; 12.7) | | 433 | | 11.5 | (8.9; 14.9) | | 485 | | 13.4 | (10.7; 16.7) | | 415 | | 12.8 | (9.9; 16.3) | | 3.1 | | 0.0913 |  | | |  |
|  | 85-89 | 558 | | 7.9 | (5.9; 10.4) | | 553 | | 8.5 | (6.5; 11.1) | | 538 | | 9.7 | (7.4; 12.5) | | 488 | | 8.4 | (6.3; 11.2) | | 0.5 | | 0.5924 |  | | |  |
|  | ≥90 | 652 | | 7.7 | (5.9; 10.0) | | 704 | | 6.0 | (4.4; 8.0) | | 636 | | 7.1 | (5.3; 9.3) | | 551 | | 5.6 | (4.0; 7.9) | | -2.0 | | 0.2807 |  | | |  |
| Females | 70-74 | 211 | | 15.2 | (11.0; 20.6) | | 237 | | 16.0 | (11.9; 21.2) | | 250 | | 16.4 | (12.3; 21.5) | | 215 | | 18.6 | (14.0; 24.3) | | 3.4 | | 0.3491 |  | | |  |
|  | 75-79 | 315 | | 10.5 | (7.6; 14.3) | | 347 | | 12.1 | (9.1; 16.0) | | 345 | | 16.2 | (12.7; 20.5) | | 319 | | 13.8 | (10.4; 18.0) | | 3.3 | | 0.0915 |  | | |  |
|  | 80-84 | 427 | | 8.2 | (6.0; 11.2) | | 467 | | 11.1 | (8.6; 14.3) | | 483 | | 11.4 | (8.9; 14.5) | | 391 | | 10.0 | (7.4; 13.3) | | 1.8 | | 0.3768 |  | | |  |
|  | 85-89 | 545 | | 6.2 | (4.5; 8.6) | | 576 | | 7.3 | (5.4; 9.7) | | 547 | | 8.4 | (6.4; 11.0) | | 535 | | 6.2 | (4.4; 8.5) | | -0.1 | | 0.8466 |  | | |  |
|  | ≥90 | 526 | | 3.8 | (2.5; 5.8) | | 592 | | 6.4 | (4.7; 8.7) | | 647 | | 4.8 | (3.4; 6.7) | | 574 | | 6.6 | (4.9; 9.0) | | 2.8 | | 0.1299 |  | | |  |
| **QI_Dipstick_:** Proportion of patients ≥70 years with incident CKD who had a dipstick-test for protein, glucose, erythrocytes, leukocytes, and nitrite | | | | | | | | | | | | | | | | | | | | | | | | | | | |  |
| Males | 70-74 | 217 | | 62.7 | (56.1; 68.8) | | 285 | | 58.2 | (52.4; 63.8) | | 312 | | 62.5 | (57.0; 67.7) | | 250 | | 60.0 | (53.8; 65.9) | | -2.7 | | 0.8679 |  | | |  |
|  | 75-79 | 340 | | 61.8 | (56.5; 66.8) | | 373 | | 61.1 | (56.1; 65.9) | | 401 | | 59.4 | (54.5; 64.0) | | 315 | | 59.0 | (53.5; 64.3) | | -2.7 | | 0.3996 |  | | |  |
|  | 80-84 | 467 | | 59.3 | (54.8; 63.7) | | 433 | | 56.6 | (51.9; 61.2) | | 485 | | 61.2 | (56.8; 65.5) | | 415 | | 55.9 | (51.1; 60.6) | | -3.4 | | 0.6310 |  | | |  |
|  | 85-89 | 558 | | 54.1 | (50.0; 58.2) | | 553 | | 58.0 | (53.9; 62.1) | | 538 | | 55.6 | (51.4; 59.7) | | 488 | | 51.6 | (47.2; 56.0) | | -2.5 | | 0.3421 |  | | |  |
|  | ≥90 | 652 | | 41.7 | (38.0; 45.5) | | 704 | | 43.2 | (39.6; 46.9) | | 636 | | 42.9 | (39.1; 46.8) | | 551 | | 41.2 | (37.2; 45.4) | | -0.5 | | 0.8682 |  | | |  |
| Females | 70-74 | 211 | | 61.1 | (54.4; 67.5) | | 237 | | 65.8 | (59.6; 71.6) | | 250 | | 64.4 | (58.3; 70.1) | | 215 | | 63.7 | (57.1; 69.9) | | 2.6 | | 0.6797 |  | | |  |
|  | 75-79 | 315 | | 58.1 | (52.6; 63.4) | | 347 | | 58.8 | (53.5; 63.8) | | 345 | | 58.6 | (53.3; 63.6) | | 319 | | 58.3 | (52.8; 63.6) | | 0.2 | | 0.9766 |  | | |  |
|  | 80-84 | 427 | | 49.6 | (44.9; 54.4) | | 467 | | 52.5 | (47.9; 57.0) | | 483 | | 52.4 | (47.9; 56.8) | | 391 | | 51.7 | (46.7; 56.6) | | 2.0 | | 0.5762 |  | | |  |
|  | 85-89 | 545 | | 36.3 | (32.4; 40.5) | | 576 | | 40.8 | (36.9; 44.9) | | 547 | | 42.8 | (38.7; 47.0) | | 535 | | 42.2 | (38.1; 46.5) | | 5.9 | | 0.0365 |  | | |  |
|  | ≥90 | 526 | | 28.7 | (25.0; 32.7) | | 592 | | 30.6 | (27.0; 34.4) | | 647 | | 29.7 | (26.3; 33.3) | | 574 | | 28.6 | (25.0; 32.4) | | -0.1 | | 0.8590 |  | | |  |
|  | | | | | | | | | | | | | | | | | | | | | | | | | | | |  |
| **QI_ACEi+ARB_:** Proportion of patients ≥70 years with CKD who had multiple (≥2) concomitant prescriptions of angiotensin-converting-enzyme (ACE) inhibitors and angiotensin II receptor blockers (ARB) over at least two quarters within three quarters after the quarter with the first simultaneous prescription *(non-recommended)*  ***Table S7*** *– continued* | | | | | | | | | | | | | | | | | | | | | | | | | | | |  |
| Males | 70-74 | 472 | | 7.0 | (5.0; 9.7) | | 499 | | 2.8 | (1.7; 4.7) | | 517 | | 2.9 | (1.8; 4.7) | | 576 | | 1.0 | (0.5; 2.3) | | -5.9 | | <0.0001 | * | | |  |
|  | 75-79 | 673 | | 4.0 | (2.8; 5.8) | | 727 | | 2.6 | (1.7; 4.0) | | 793 | | 2.1 | (1.3; 3.4) | | 890 | | 1.3 | (0.8; 2.3) | | -2.7 | | 0.0008 | * | | |  |
|  | 80-84 | 908 | | 3.2 | (2.2; 4.5) | | 1,055 | | 1.8 | (1.2; 2.8) | | 1,153 | | 1.3 | (0.8; 2.1) | | 1,232 | | 1.6 | (1.1; 2.5) | | -1.6 | | 0.0099 |  | | |  |
|  | 85-89 | 1,271 | | 2.1 | (1.5; 3.1) | | 1,404 | | 1.1 | (0.7; 1.8) | | 1,558 | | 0.9 | (0.5; 1.5) | | 1,690 | | 0.6 | (0.3; 1.1) | | -1.5 | | 0.0001 | * | | |  |
|  | ≥90 | 1,418 | | 1.6 | (1.0; 2.3) | | 1,610 | | 0.6 | (0.3; 1.1) | | 1,906 | | 0.3 | (0.1; 0.7) | | 2,104 | | 0.1 | (0.0; 0.4) | | -1.4 | | <0.0001 | * | | |  |
| Females | 70-74 | 363 | | 4.7 | (2.9; 7.4) | | 381 | | 3.1 | (1.8; 5.4) | | 432 | | 1.4 | (0.6; 3.0) | | 474 | | 1.9 | (1.0; 3.6) | | -2.8 | | 0.0060 | * | | |  |
|  | 75-79 | 504 | | 4.0 | (2.6; 6.0) | | 614 | | 3.9 | (2.6; 5.8) | | 641 | | 1.7 | (1.0; 3.0) | | 768 | | 1.4 | (0.8; 2.5) | | -2.5 | | 0.0006 | * | | |  |
|  | 80-84 | 681 | | 2.3 | (1.5; 3.8) | | 809 | | 2.0 | (1.2; 3.2) | | 953 | | 1.4 | (0.8; 2.3) | | 1,134 | | 1.4 | (0.9; 2.3) | | -0.9 | | 0.0943 |  | | |  |
|  | 85-89 | 921 | | 2.7 | (1.8; 4.0) | | 1,093 | | 2.5 | (1.7; 3.6) | | 1,268 | | 1.1 | (0.7; 1.8) | | 1,445 | | 1.1 | (0.7; 1.8) | | -1.6 | | 0.0004 | * | | |  |
|  | ≥90 | 1,010 | | 1.3 | (0.8; 2.2) | | 1,232 | | 0.3 | (0.1; 0.8) | | 1,542 | | 0.6 | (0.4; 1.2) | | 1,745 | | 0.6 | (0.4; 1.1) | | -0.7 | | 0.1981 |  | | |  |
| **QI_NSAID_:** Proportion of patients ≥70 years with CKD stage 4-5 (eGFR <30 ml/min/1.73m²) for whom NSAIDs (nonsteroidal anti-inflammatory drugs) were prescribed *(non-recommended)* | | | | | | | | | | | | | | | | | | | | | | | | | | | |  |
| Males | 70-74 | 62 | | 1.6 | (0.3; 8.6) | | 75 | | 2.7 | (0.7; 9.2) | | 65 | | 1.5 | (0.3; 8.2) | | 91 | | 0.0 | (0.0; 4.1) | | -1.6 | | 0.2605 |  | | |  |
|  | 75-79 | 84 | | 3.6 | (1.2; 10.0) | | 103 | | 2.9 | (1.0; 8.2) | | 114 | | 1.8 | (0.5; 6.2) | | 112 | | 1.8 | (0.5; 6.3) | | -1.8 | | 0.3544 |  | | |  |
|  | 80-84 | 115 | | 4.3 | (1.9; 9.8) | | 179 | | 2.8 | (1.2; 6.4) | | 205 | | 3.9 | (2.0; 7.5) | | 190 | | 4.2 | (2.1; 8.1) | | -0.1 | | 0.8098 |  | | |  |
|  | 85-89 | 183 | | 9.8 | (6.3; 15.0) | | 238 | | 2.9 | (1.4; 5.9) | | 283 | | 3.5 | (1.9; 6.4) | | 330 | | 2.1 | (1.0; 4.3) | | -7.7 | | 0.0003 | * | | |  |
|  | ≥90 | 188 | | 9.6 | (6.1; 14.6) | | 267 | | 4.9 | (2.9; 8.2) | | 356 | | 3.4 | (1.9; 5.8) | | 431 | | 4.6 | (3.0; 7.1) | | -4.9 | | 0.0270 |  | | |  |
| Females | 70-74 | 62 | | 1.6 | (0.3; 8.6) | | 65 | | 7.7 | (3.3; 16.8) | | 71 | | 8.5 | (3.9; 17.2) | | 69 | | 1.4 | (0.3; 7.8) | | -0.2 | | 0.9678 |  | | |  |
|  | 75-79 | 72 | | 5.6 | (2.2; 13.4) | | 113 | | 5.3 | (2.5; 11.1) | | 105 | | 0.0 | (0.0; 3.5) | | 126 | | 2.4 | (0.8; 6.8) | | -3.2 | | 0.0717 |  | | |  |
|  | 80-84 | 110 | | 5.5 | (2.5; 11.4) | | 164 | | 4.3 | (2.1; 8.5) | | 193 | | 2.6 | (1.1; 5.9) | | 214 | | 2.3 | (1.0; 5.4) | | -3.1 | | 0.1007 |  | | |  |
|  | 85-89 | 141 | | 9.9 | (6.0; 16.0) | | 232 | | 5.6 | (3.3; 9.3) | | 274 | | 4.4 | (2.5; 7.5) | | 287 | | 1.7 | (0.7; 4.0) | | -8.2 | | 0.0002 | * | | |  |
|  | ≥90 | 122 | | 12.3 | (7.6; 19.3) | | 190 | | 4.2 | (2.1; 8.1) | | 290 | | 4.5 | (2.6; 7.5) | | 384 | | 4.7 | (3.0; 7.3) | | -7.6 | | 0.0198 |  | | |  |
|  | | | | | | | | | | | | | | | | | | | | | | | | | | | | |
| ***Metamizole:*** Proportion of patients ≥70 years with CKD stage 4-5 (eGFR <30 ml/min/1.73m²) for whom Metamizole was prescribed *(exploratory)*  ***Table S7*** *– continued* | | | | | | | | | | | | | | | | | | | | | | | | | | | |  |
| Males | 70-74 | 62 | | 27.4 | (17.9; 39.6) | | 75 | | 37.3 | (27.3; 48.6) | | 65 | | 40.0 | (29.0; 52.1) | | 91 | | 38.5 | (29.1; 48.7) | | 11.0 | | n.a. |  | | |  |
|  | 75-79 | 84 | | 35.7 | (26.3; 46.4) | | 103 | | 32.0 | (23.8; 41.6) | | 114 | | 38.6 | (30.2; 47.8) | | 112 | | 42.0 | (33.2; 51.2) | | 6.2 | | n.a. |  | | |  |
|  | 80-84 | 115 | | 26.1 | (18.9; 34.8) | | 179 | | 35.8 | (29.1; 43.0) | | 205 | | 33.2 | (27.1; 39.9) | | 190 | | 32.1 | (25.9; 39.0) | | 6.0 | | n.a. |  | | |  |
|  | 85-89 | 183 | | 38.8 | (32.0; 46.0) | | 238 | | 40.3 | (34.3; 46.7) | | 283 | | 42.8 | (37.1; 48.6) | | 330 | | 45.2 | (39.9; 50.5) | | 6.4 | | n.a. |  | | |  |
|  | ≥90 | 188 | | 36.2 | (29.6; 43.3) | | 267 | | 37.8 | (32.2; 43.8) | | 356 | | 41.9 | (36.8; 47.0) | | 431 | | 45.2 | (40.6; 50.0) | | 9.1 | | n.a. |  | | |  |
| Females | 70-74 | 62 | | 33.9 | (23.3; 46.3) | | 65 | | 52.3 | (40.4; 64.0) | | 71 | | 46.5 | (35.4; 58.0) | | 69 | | 52.2 | (40.6; 63.5) | | 18.3 | | n.a. |  | | |  |
|  | 75-79 | 72 | | 45.8 | (34.8; 57.3) | | 113 | | 47.8 | (38.8; 56.9) | | 105 | | 56.2 | (46.6; 65.3) | | 126 | | 50.8 | (42.2; 59.4) | | 5.0 | | n.a. |  | | |  |
|  | 80-84 | 110 | | 46.4 | (37.3; 55.6) | | 164 | | 50.0 | (42.4; 57.6) | | 193 | | 43.0 | (36.2; 50.1) | | 214 | | 45.3 | (38.8; 52.0) | | -1.0 | | n.a. |  | | |  |
|  | 85-89 | 141 | | 46.8 | (38.8; 55.0) | | 232 | | 50.0 | (43.6; 56.4) | | 274 | | 47.8 | (42.0; 53.7) | | 287 | | 53.7 | (47.9; 59.3) | | 6.9 | | n.a. |  | | |  |
|  | ≥90 | 122 | | 50.0 | (41.3; 58.7) | | 190 | | 42.6 | (35.8; 49.7) | | 290 | | 57.6 | (51.8; 63.1) | | 384 | | 55.2 | (50.2; 60.1) | | 5.2 | | n.a. |  | | |  |
| *Abbreviations:* QI: quality indicator. 95%‑CI: 95% confidence intervals. Δ: Difference from 2012 to 2018. At risk: Number of persons at risk (denominator) for a certain QI. * Statistically significant for $p<0.0083$ (after Bonferroni-correction).  ^a^ Time trends were tested using the Cochran-Armitage test with Bonferroni correction. | | | | | | | | | | | | | | | | | | | | | | | | | | | |  |


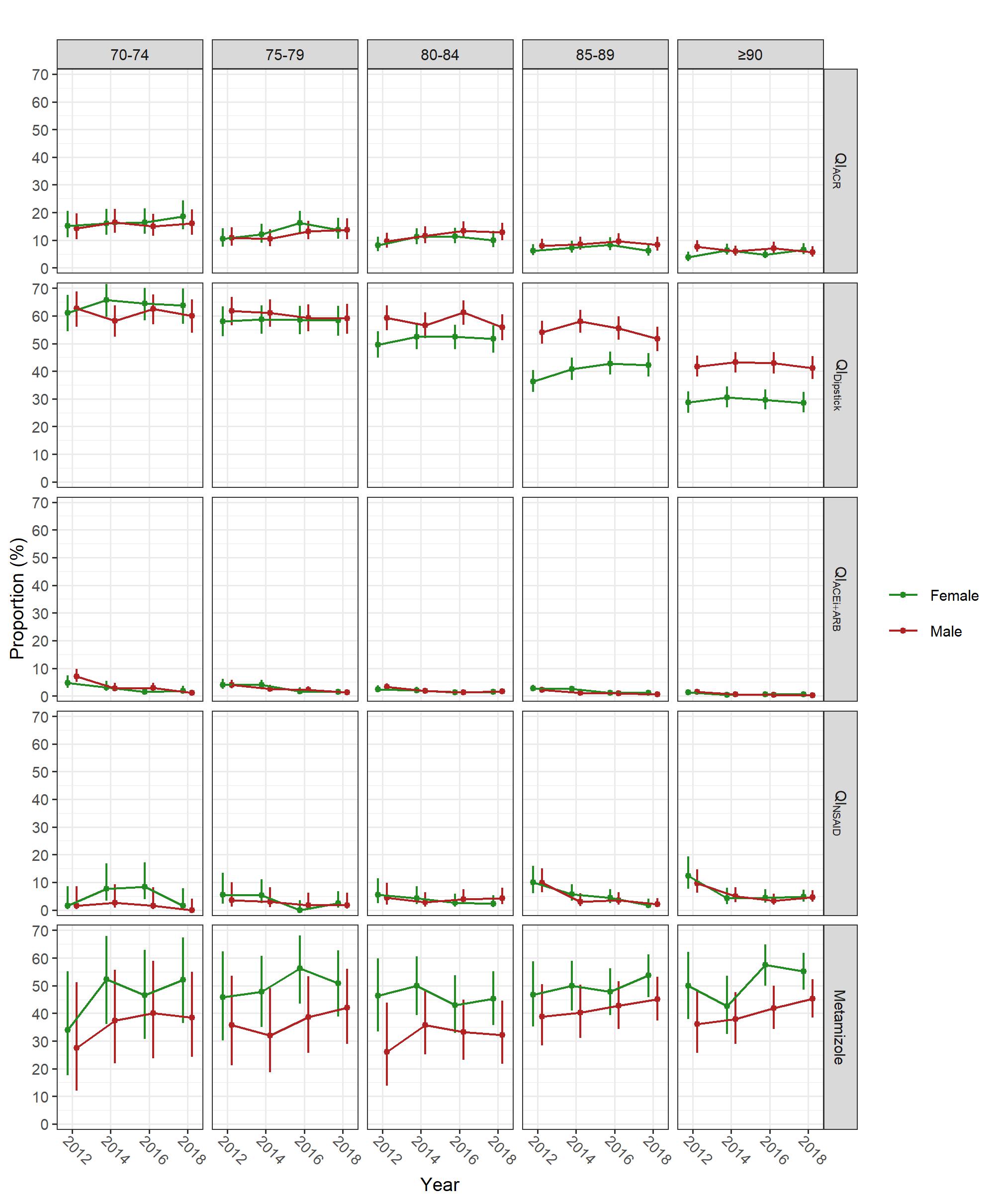


Figure S3 – Quality indicators (QI) for the outpatient healthcare service quality for patients with CKD, stratified by sex and age. Vertical lines represent 95% confidence intervals. Values are interpolated between consecutive years for graphical display.


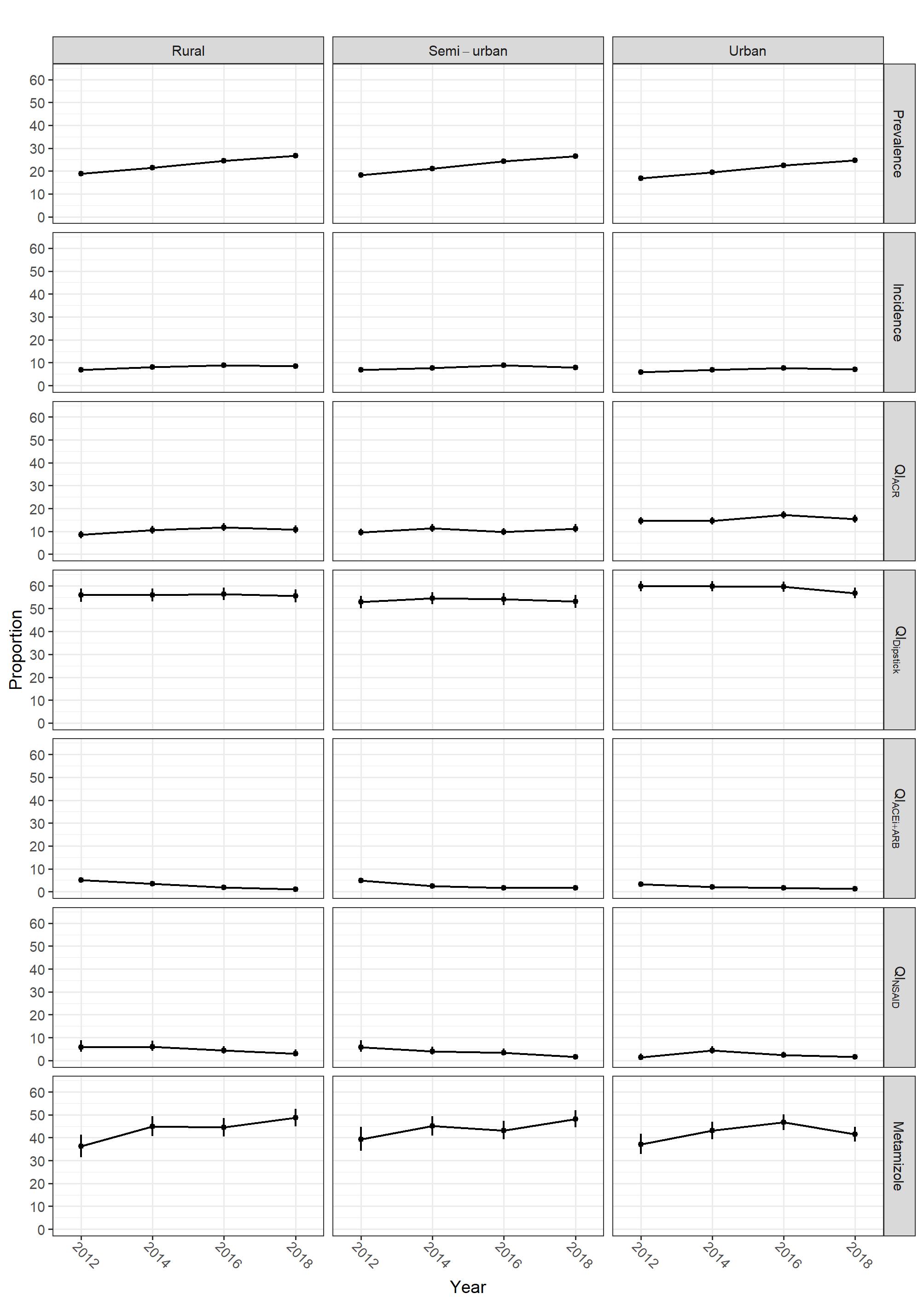


Figure S4 – Prevalence, incidence, and quality indicators (QI) for the outpatient healthcare service quality for patients with CKD, stratified by region of residence. Vertical lines represent 95% confidence intervals. Values are interpolated between consecutive years for graphical display.

Table S8 – Prevalence, incidence, and quality indicators (QI) for the outpatient healthcare service quality for patients with CKD, stratified by healthcare service provider.

| **QI** | **Healthcare service provider** |  | **2012** | | |  |  | **2014** | | |  |  | **2016** | | |  |  | **2018** | | |  | Δ |
| --- | --- | --- | --- | --- | --- | --- | --- | --- | --- | --- | --- | --- | --- | --- | --- | --- | --- | --- | --- | --- | --- | --- |
|  |  | At risk | | % | (95%‑CI) | | At risk | | % | (95%‑CI) | | At risk | | % | (95%‑CI) | | At risk | | % | (95%‑CI) | |  |
| **Prevalence –** CKD stage G3-5 | Total | 61,970 | | 17.8 | (17.5; 18.1) | | 61,986 | | 20.5 | (20.2; 20.8) | | 62,018 | | 23.5 | (23.1; 23.8) | | 61,994 | | 25.7 | (25.4; 26.1) | | +8.0 |
|  | Kidney specialist | 61,970 | | 4.4 | (4.3; 4.6) | | 61,986 | | 4.9 | (4.7; 5.1) | | 62,018 | | 5.2 | (5.0; 5.4) | | 61,994 | | 5.4 | (5.2; 5.6) | | +1.0 |
|  | GP + hospital | 61,970 | | 2.3 | (2.2; 2.5) | | 61,986 | | 3.2 | (3.1; 3.4) | | 62,018 | | 3.6 | (3.4; 3.7) | | 61,994 | | 3.8 | (3.7; 4.0) | | +1.5 |
|  | GP only | 61,970 | | 7.1 | (6.9; 7.3) | | 61,986 | | 8.0 | (7.8; 8.2) | | 62,018 | | 10.1 | (9.9; 10.4) | | 61,994 | | 11.5 | (11.3; 11.8) | | +4.4 |
|  | Hospital | 61,970 | | 3.3 | (3.2; 3.5) | | 61,986 | | 3.7 | (3.5; 3.8) | | 62,018 | | 3.7 | (3.6; 3.9) | | 61,994 | | 4.0 | (3.9; 4.2) | | +0.7 |
|  | Other | 61,970 | | 0.6 | (0.6; 0.7) | | 61,986 | | 0.7 | (0.6; 0.7) | | 62,018 | | 0.9 | (0.8; 0.9) | | 61,994 | | 1.0 | (0.9; 1.1) | | +0.4 |
| **Prevalence –** CKD stage G3 | Total | 61,970 | | 8.2 | (8.0; 8.5) | | 61,986 | | 10.7 | (10.4; 10.9) | | 62,018 | | 13.7 | (13.4; 13.9) | | 61,994 | | 15.7 | (15.4; 16.0) | | +7.4 |
|  | Kidney specialist | 61,970 | | 1.8 | (1.7; 1.9) | | 61,986 | | 2.3 | (2.1; 2.4) | | 62,018 | | 2.5 | (2.4; 2.6) | | 61,994 | | 2.7 | (2.6; 2.9) | | +0.9 |
|  | GP + hospital | 61,970 | | 0.6 | (0.6; 0.7) | | 61,986 | | 1.1 | (1.0; 1.2) | | 62,018 | | 1.4 | (1.3; 1.5) | | 61,994 | | 1.7 | (1.6; 1.8) | | +1.1 |
|  | GP only | 61,970 | | 2.1 | (2.0; 2.2) | | 61,986 | | 3.2 | (3.1; 3.3) | | 62,018 | | 5.5 | (5.3; 5.7) | | 61,994 | | 6.8 | (6.6; 7.0) | | +4.6 |
|  | Hospital | 61,970 | | 3.5 | (3.3; 3.6) | | 61,986 | | 3.9 | (3.7; 4.0) | | 62,018 | | 3.7 | (3.6; 3.9) | | 61,994 | | 3.9 | (3.8; 4.1) | | +0.4 |
|  | Other | 61,970 | | 0.2 | (0.2; 0.2) | | 61,986 | | 0.3 | (0.2; 0.3) | | 62,018 | | 0.5 | (0.4; 0.5) | | 61,994 | | 0.6 | (0.5; 0.6) | | +0.4 |
| **Prevalence –** CKD stage G4-5 | Total | 61,970 | | 2.8 | (2.7; 3.0) | | 61,986 | | 3.6 | (3.4; 3.7) | | 62,018 | | 4.0 | (3.9; 4.2) | | 61,994 | | 4.4 | (4.2; 4.6) | | +1.6 |
|  | Kidney specialist | 61,970 | | 1.0 | (1.0; 1.1) | | 61,986 | | 1.2 | (1.1; 1.3) | | 62,018 | | 1.3 | (1.2; 1.4) | | 61,994 | | 1.4 | (1.4; 1.5) | | +0.4 |
|  | GP + hospital | 61,970 | | 0.1 | (0.1; 0.2) | | 61,986 | | 0.3 | (0.3; 0.4) | | 62,018 | | 0.4 | (0.3; 0.4) | | 61,994 | | 0.4 | (0.3; 0.4) | | +0.2 |
|  | GP only | 61,970 | | 0.5 | (0.5; 0.6) | | 61,986 | | 0.8 | (0.7; 0.8) | | 62,018 | | 1.1 | (1.0; 1.2) | | 61,994 | | 1.2 | (1.2; 1.3) | | +0.7 |
|  | Hospital | 61,970 | | 1.0 | (1.0; 1.1) | | 61,986 | | 1.1 | (1.1; 1.2) | | 62,018 | | 1.1 | (1.0; 1.2) | | 61,994 | | 1.1 | (1.0; 1.2) | | +0.1 |
|  | Other | 61,970 | | 0.1 | (0.1; 0.2) | | 61,986 | | 0.1 | (0.1; 0.2) | | 62,018 | | 0.2 | (0.2; 0.2) | | 61,994 | | 0.2 | (0.2; 0.2) | | +0.1 |
| **Incidence –** CKD stage G3-5 | Total | 50,032 | | 6.4 | (6.2; 6.6) | | 48,378 | | 7.4 | (7.2; 7.6) | | 46,731 | | 8.3 | (8.0; 8.5) | | 44,903 | | 7.6 | (7.4; 7.9) | | +1.3 |
|  | Kidney specialist | 50,032 | | 0.7 | (0.6; 0.8) | | 48,378 | | 0.8 | (0.7; 0.9) | | 46,731 | | 0.8 | (0.7; 0.9) | | 44,903 | | 0.6 | (0.6; 0.7) | | -0.1 |
|  | GP + hospital | 50,032 | | 0.7 | (0.7; 0.8) | | 48,378 | | 1.0 | (0.9; 1.1) | | 46,731 | | 0.9 | (0.8; 1.0) | | 44,903 | | 0.8 | (0.7; 0.9) | | +0.1 |
|  | GP only | 50,032 | | 1.8 | (1.6; 1.9) | | 48,378 | | 2.0 | (1.9; 2.1) | | 46,731 | | 2.7 | (2.6; 2.9) | | 44,903 | | 2.0 | (1.9; 2.1) | | +0.2 |
|  | Hospital | 50,032 | | 3.0 | (2.8; 3.1) | | 48,378 | | 3.3 | (3.2; 3.5) | | 46,731 | | 3.4 | (3.2; 3.6) | | 44,903 | | 3.8 | (3.7; 4.0) | | +0.9 |
|  | Other | 50,032 | | 0.3 | (0.2; 0.3) | | 48,378 | | 0.3 | (0.3; 0.4) | | 46,731 | | 0.4 | (0.4; 0.5) | | 44,903 | | 0.4 | (0.3; 0.4) | | +0.1 |
| **Incidence** **–** CKD stage G3 | Total | 56,093 | | 3.3 | (3.1; 3.4) | | 54,095 | | 4.1 | (4.0; 4.3) | | 52,371 | | 5.0 | (4.8; 5.2) | | 49,996 | | 4.6 | (4.4; 4.7) | | +1.3 |
|  | Kidney specialist | 56,093 | | 0.3 | (0.2; 0.3) | | 54,095 | | 0.4 | (0.4; 0.5) | | 52,371 | | 0.5 | (0.4; 0.5) | | 49,996 | | 0.3 | (0.3; 0.4) | | +0.1 |
|  | GP + hospital | 56,093 | | 0.2 | (0.2; 0.2) | | 54,095 | | 0.4 | (0.3; 0.4) | | 52,371 | | 0.4 | (0.3; 0.4) | | 49,996 | | 0.4 | (0.3; 0.4) | | +0.2 |
|  | GP only | 56,093 | | 0.5 | (0.4; 0.5) | | 54,095 | | 0.8 | (0.7; 0.9) | | 52,371 | | 1.6 | (1.5; 1.7) | | 49,996 | | 1.0 | (0.9; 1.0) | | +0.5 |
|  | Hospital | 56,093 | | 2.2 | (2.1; 2.4) | | 54,095 | | 2.5 | (2.3; 2.6) | | 52,371 | | 2.4 | (2.3; 2.5) | | 49,996 | | 2.8 | (2.6; 2.9) | | +0.5 |
|  | Other | 56,093 | | 0.1 | (0.0; 0.1) | | 54,095 | | 0.1 | (0.0; 0.1) | | 52,371 | | 0.2 | (0.1; 0.2) | | 49,996 | | 0.2 | (0.1; 0.2) | | +0.1 |
| **Incidence –** CKD stage G4-5 | Total | 60,325 | | 1.5 | (1.4; 1.6) | | 59,820 | | 1.7 | (1.6; 1.8) | | 59,517 | | 1.8 | (1.7; 1.9) | | 59,214 | | 1.8 | (1.7; 1.9) | | +0.3 |
|  | Kidney specialist | 60,325 | | 0.3 | (0.3; 0.4) | | 59,820 | | 0.3 | (0.3; 0.3) | | 59,517 | | 0.3 | (0.3; 0.4) | | 59,214 | | 0.4 | (0.3; 0.4) | | +0.0 |
|  | GP + hospital | 60,325 | | 0.1 | (0.1; 0.1) | | 59,820 | | 0.1 | (0.1; 0.1) | | 59,517 | | 0.1 | (0.1; 0.2) | | 59,214 | | 0.1 | (0.1; 0.2) | | +0.1 |
|  | GP only | 60,325 | | 0.2 | (0.2; 0.3) | | 59,820 | | 0.3 | (0.2; 0.3) | | 59,517 | | 0.3 | (0.3; 0.4) | | 59,214 | | 0.3 | (0.3; 0.4) | | +0.1 |
|  | Hospital | 60,325 | | 0.8 | (0.7; 0.9) | | 59,820 | | 0.9 | (0.8; 1.0) | | 59,517 | | 0.9 | (0.8; 1.0) | | 59,214 | | 0.9 | (0.8; 1.0) | | +0.1 |
|  | Other | 60,325 | | 0.0 | (0.0; 0.1) | | 59,820 | | 0.1 | (0.0; 0.1) | | 59,517 | | 0.1 | (0.1; 0.1) | | 59,214 | | 0.1 | (0.1; 0.1) | | +0.0 |
|  |  |  | |  |  | |  | |  |  | |  | |  |  | |  | |  |  | |  |
| **QI_ACR_**  ***Table S8*** *– continued* | Total | 4,258 | | 11.4 | (10.5; 12.4) | | 4,567 | | 12.6 | (11.6; 13.5) | | 4,644 | | 13.5 | (12.5; 14.5) | | 4,053 | | 12.9 | (11.9; 14.0) | | +1.5 |
|  | Kidney specialist | 412 | | 40.7 | (36.0; 45.5) | | 419 | | 44.8 | (40.1; 49.6) | | 427 | | 46.4 | (41.7; 51.1) | | 295 | | 58.0 | (52.3; 63.5) | | +17.4 |
|  | GP + hospital | 570 | | 9.0 | (6.9; 11.6) | | 655 | | 12.1 | (9.8; 14.8) | | 600 | | 12.1 | (9.8; 15.0) | | 476 | | 13.5 | (10.7; 16.9) | | +4.5 |
|  | GP only | 1,059 | | 14.9 | (12.9; 17.2) | | 1,145 | | 17.1 | (15.0; 19.3) | | 1,407 | | 18.4 | (16.5; 20.5) | | 979 | | 18.1 | (15.8; 20.6) | | +3.2 |
|  | Hospital | 2,058 | | 1.6 | (1.2; 2.3) | | 2,190 | | 0.4 | (0.2; 0.8) | | 2,004 | | 1.2 | (0.8; 1.8) | | 2,130 | | 1.1 | (0.8; 1.7) | | -0.5 |
|  | Other | 159 | | 20.8 | (15.2; 27.8) | | 158 | | 19.7 | (14.2; 26.6) | | 206 | | 13.8 | (9.8; 19.2) | | 173 | | 15.9 | (11.2; 22.1) | | -4.9 |
| **QI_Dipstick_** | Total | 4,258 | | 56.6 | (55.1; 58.1) | | 4,567 | | 57.2 | (55.8; 58.6) | | 4,644 | | 57.0 | (55.6; 58.5) | | 4,053 | | 55.4 | (53.8; 56.9) | | -1.2 |
|  | Kidney specialist | 412 | | 91.4 | (88.3; 93.7) | | 419 | | 89.7 | (86.5; 92.3) | | 427 | | 92.3 | (89.4; 94.5) | | 295 | | 90.4 | (86.5; 93.3) | | -0.9 |
|  | GP + hospital | 570 | | 49.9 | (45.8; 54.0) | | 655 | | 53.9 | (50.0; 57.6) | | 600 | | 47.3 | (43.3; 51.3) | | 476 | | 41.1 | (36.8; 45.6) | | -8.8 |
|  | GP only | 1,059 | | 62.1 | (59.1; 64.9) | | 1,145 | | 58.6 | (55.7; 61.4) | | 1,407 | | 60.5 | (57.9; 63.0) | | 979 | | 61.3 | (58.2; 64.3) | | -0.8 |
|  | Hospital | 2,058 | | 45.0 | (42.8; 47.1) | | 2,190 | | 47.7 | (45.6; 49.7) | | 2,004 | | 46.8 | (44.6; 49.0) | | 2,130 | | 47.4 | (45.2; 49.5) | | +2.4 |
|  | Other | 159 | | 63.3 | (55.6; 70.4) | | 158 | | 72.2 | (64.7; 78.6) | | 206 | | 59.3 | (52.5; 65.8) | | 173 | | 64.3 | (57.0; 71.1) | | +1.0 |
| **QI_ACEi+ARB_** | Total | 8,221 | | 4.2 | (3.8; 4.7) | | 9,424 | | 2.7 | (2.4; 3.1) | | 10,763 | | 1.7 | (1.5; 1.9) | | 12,058 | | 1.3 | (1.2; 1.6) | | -2.9 |
|  | Kidney specialist | 2,285 | | 5.7 | (4.8; 6.7) | | 2,464 | | 2.8 | (2.2; 3.6) | | 2,682 | | 2.0 | (1.6; 2.7) | | 2,784 | | 2.0 | (1.5; 2.6) | | -3.7 |
|  | GP + hospital | 1,401 | | 2.8 | (2.1; 3.8) | | 1,794 | | 3.9 | (3.1; 4.9) | | 2,080 | | 2.1 | (1.5; 2.8) | | 2,227 | | 1.6 | (1.2; 2.2) | | -1.2 |
|  | GP only | 3,643 | | 3.7 | (3.1; 4.3) | | 4,162 | | 2.4 | (2.0; 2.9) | | 4,915 | | 1.5 | (1.2; 1.9) | | 5,874 | | 1.1 | (0.9; 1.4) | | -2.6 |
|  | Hospital | 653 | | 3.5 | (2.3; 5.2) | | 744 | | 2.3 | (1.4; 3.6) | | 798 | | 0.6 | (0.2; 1.4) | | 793 | | 0.8 | (0.3; 1.6) | | -2.7 |
|  | Other | 239 | | 2.7 | (1.3; 5.6) | | 260 | | 1.2 | (0.4; 3.4) | | 288 | | 1.8 | (0.8; 4.1) | | 380 | | 0.9 | (0.3; 2.5) | | -1.8 |
| **QI_NSAID_** | Total | 1,139 | | 4.3 | (3.2; 5.6) | | 1,626 | | 4.6 | (3.7; 5.8) | | 1,956 | | 3.1 | (2.4; 3.9) | | 2,234 | | 2.1 | (1.6; 2.8) | | -2.2 |
|  | Kidney specialist | 534 | | 2.8 | (1.7; 4.6) | | 678 | | 3.6 | (2.5; 5.3) | | 743 | | 1.7 | (1.0; 2.9) | | 856 | | 1.8 | (1.1; 2.9) | | -1.0 |
|  | GP + hospital | 79 | | 0.7 | (0.1; 5.8) | | 205 | | 5.1 | (2.9; 9.1) | | 224 | | 1.5 | (0.5; 4.1) | | 254 | | 2.6 | (1.2; 5.4) | | +1.9 |
|  | GP only | 249 | | 2.9 | (1.5; 5.9) | | 467 | | 2.9 | (1.7; 4.9) | | 694 | | 3.1 | (2.0; 4.6) | | 807 | | 1.4 | (0.8; 2.5) | | -1.5 |
|  | Hospital | 226 | | 7.7 | (4.9; 12.0) | | 225 | | 9.0 | (5.9; 13.4) | | 208 | | 10.1 | (6.7; 14.9) | | 222 | | 5.4 | (3.1; 9.2) | | -2.3 |
|  | Other | 51 | | 9.8 | (4.3; 21.0) | | 51 | | 25.6 | (15.7; 39.0) | | 87 | | 2.8 | (0.9; 8.8) | | 95 | | 3.0 | (1.0; 8.6) | | -6.8 |
| **Metamizole** | Total | 1,139 | | 37.3 | (34.5; 40.1) | | 1,626 | | 43.8 | (41.4; 46.2) | | 1,956 | | 45.1 | (42.9; 47.3) | | 2,234 | | 45.7 | (43.6; 47.7) | | +8.4 |
|  | Kidney specialist | 534 | | 38.5 | (34.4; 42.7) | | 678 | | 45.6 | (41.9; 49.4) | | 743 | | 44.0 | (40.5; 47.6) | | 856 | | 50.4 | (47.1; 53.7) | | +11.9 |
|  | GP + hospital | 79 | | 53.0 | (42.1; 63.6) | | 205 | | 53.4 | (46.6; 60.1) | | 224 | | 61.9 | (55.3; 68.0) | | 254 | | 43.7 | (37.7; 49.8) | | -9.3 |
|  | GP only | 249 | | 31.7 | (26.2; 37.7) | | 467 | | 32.8 | (28.7; 37.1) | | 694 | | 43.9 | (40.2; 47.6) | | 807 | | 37.8 | (34.5; 41.2) | | +6.2 |
|  | Hospital | 226 | | 46.9 | (40.5; 53.4) | | 225 | | 50.8 | (44.3; 57.2) | | 208 | | 43.1 | (36.6; 49.9) | | 222 | | 50.4 | (43.9; 56.9) | | +3.5 |
|  | Other | 51 | | 8.0 | (3.2; 18.7) | | 51 | | 19.6 | (11.0; 32.5) | | 87 | | 42.1 | (32.2; 52.6) | | 95 | | 40.4 | (31.1; 50.4) | | +32.4 |
| *Abbreviations:* QI: quality indicator. 95%‑CI: 95% confidence intervals. Δ: Difference from 2012 to 2018. At risk: Number of persons at risk (denominator) for a certain QI. GP: General practitioner.  *Notes:* Quality indicators (QI) were defined to assess outpatient healthcare service quality and reflect only procedures performed in the outpatient setting. The group “hospital” includes patients who received their CKD diagnosis (either prevalence or incidence) in a hospital and were not seen by a kidney specialist or a general practitioner (GP) (see Table S4). Diagnostic procedures, such as ACR or dipstick testing, performed in-hospital cannot be identified in claims data and are therefore not included in the QI estimates presented here. | | | | | | | | | | | | | | | | | | | | | | |


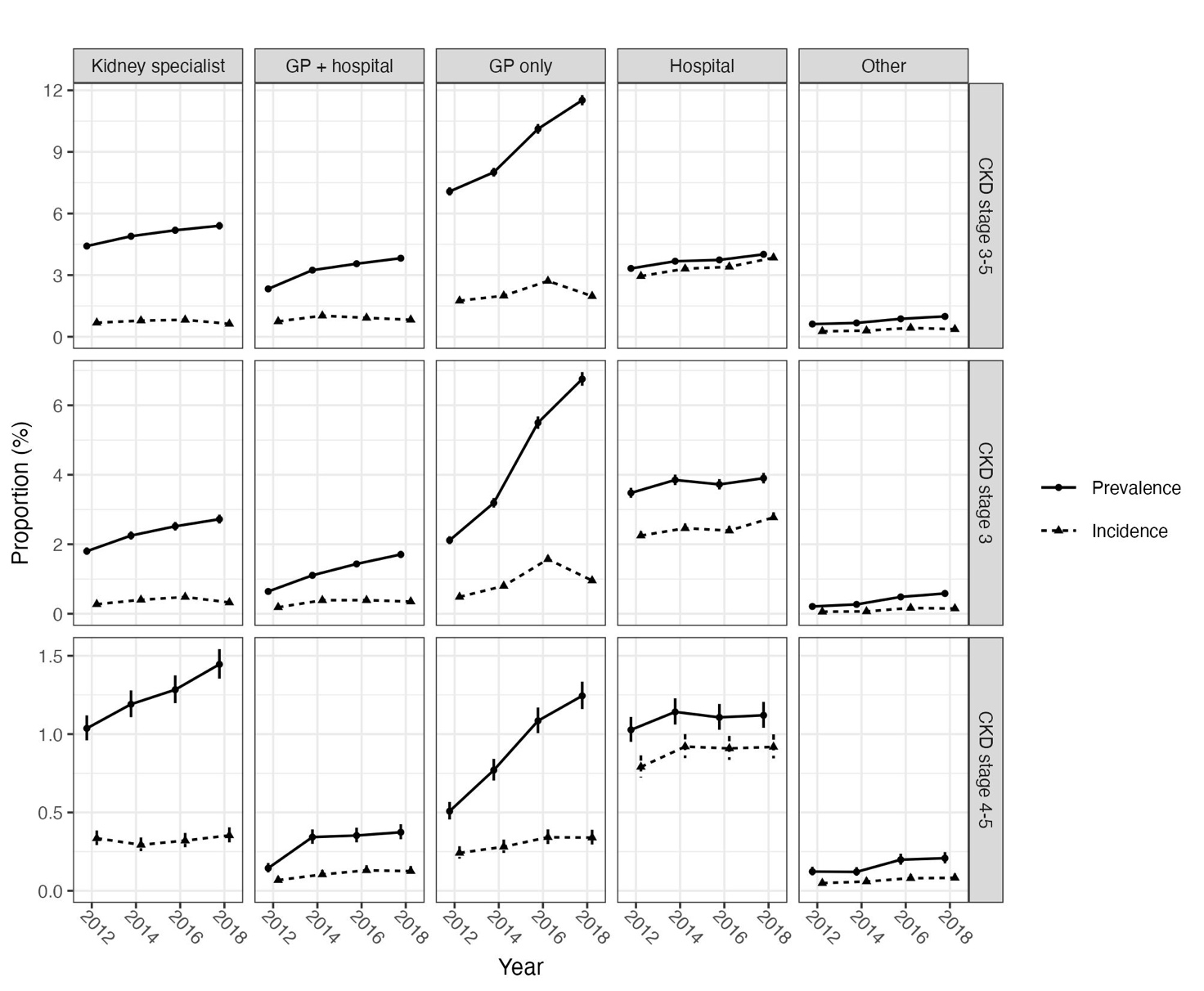


Figure S5 – Prevalence and incidence of CKD, stratified by healthcare service provider. Vertical lines represent 95% confidence intervals. Values are interpolated between consecutive years and point estimates dodged around the x-axis for graphical display.

Abbreviations: CKD: Chronic kidney disease; GP: General practitioner.


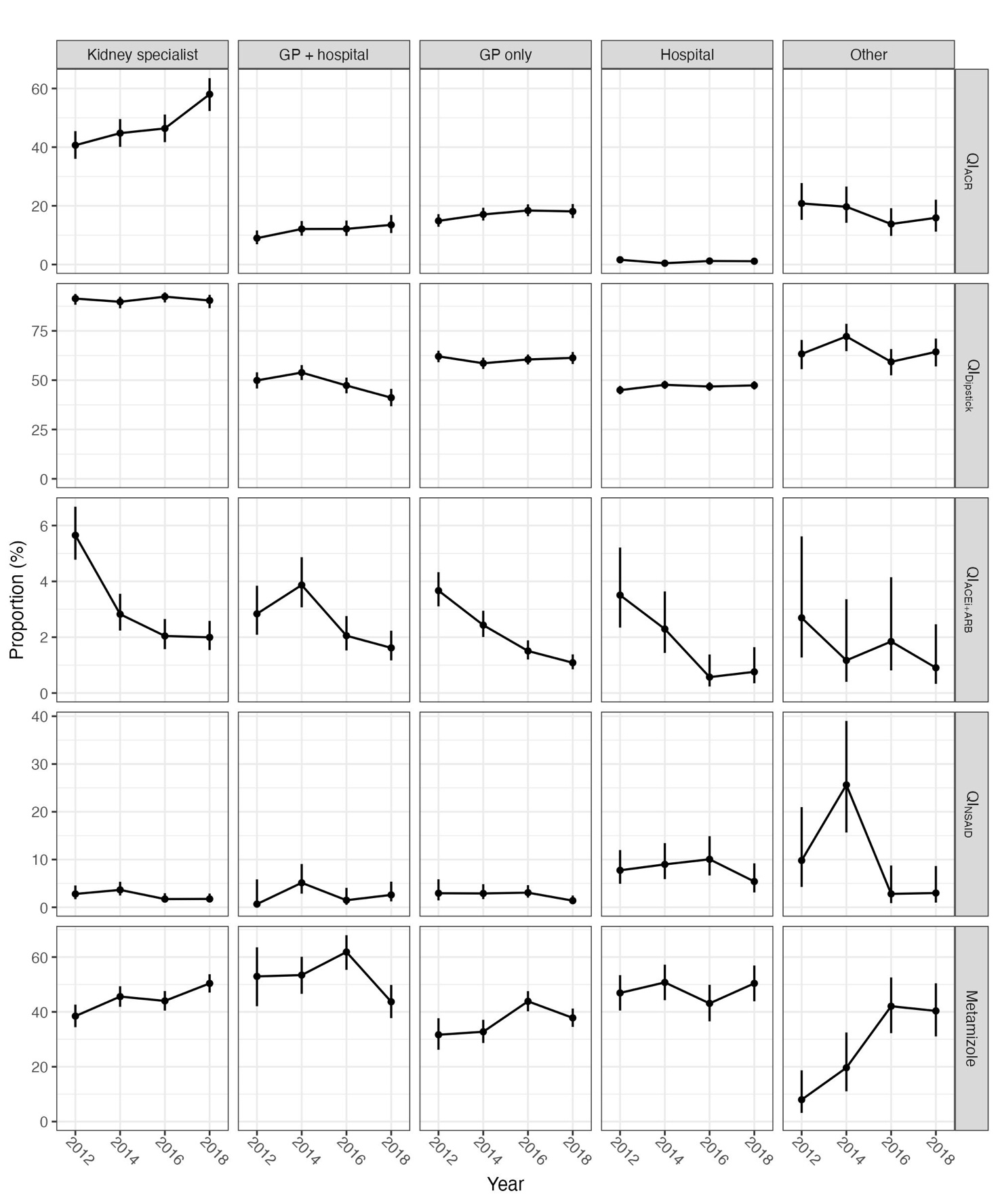


Figure S6 – Quality indicators (QI) for the outpatient healthcare service quality for patients with CKD, stratified by healthcare service provider. Vertical lines represent 95% confidence intervals. Values are interpolated between consecutive years for graphical display. Abbreviation: ACR: albumin-to-creatinine ratio; GP: General practitioner.

*Note: Quality indicators (QI) were defined to assess outpatient healthcare service quality, reflecting only procedures performed in the outpatient setting. The “hospital” group includes patients who received their CKD diagnosis (either prevalence or incidence) during hospitalization and were not seen by a kidney specialist or a general practitioner (GP) (see Table S4). Diagnostic procedures, such as ACR or dipstick testing, performed in-hospital identified in claims data and are therefore not included in the QI estimates presented here.*

Table S9 – STROBE checklist

|  | **Item No.** | **Recommendation** | **Section, paragraph** |
| --- | --- | --- | --- |
| **Title and abstract** | 1 | (a) Indicate the study’s design with a commonly used term in the title or the abstract | Abstract – Methods |
|  |  | (b) Provide in the abstract an informative and balanced summary of what was done and what was found | Abstract – Methods, Results, and Conclusion |
| **Introduction** |  |  |  |
| Background/rationale | 2 | Explain the scientific background and rationale for the investigation being reported | Introduction – Paragraphs 2­–3 |
| Objectives | 3 | State specific objectives, including any prespecified hypotheses | Introduction – Paragraph 5 |
| **Methods** |  |  |  |
| Study design | 4 | Present key elements of study design early in the paper | Methods – Paragraph 1 |
| Setting | 5 | Describe the setting, locations, and relevant dates, including periods of recruitment, exposure, follow-up, and data collection | Methods – Paragraphs 1–3 |
| Participants | 6 | (*a*) *Cohort study*—Give the eligibility criteria, and the sources and methods of selection of participants. Describe methods of follow-up  *Case-control study*—Give the eligibility criteria, and the sources and methods of case ascertainment and control selection. Give the rationale for the choice of cases and controls  *Cross-sectional study*—Give the eligibility criteria, and the sources and methods of selection of participants | Methods – Paragraphs 1 and 3 |
|  |  | (*b*) *Cohort study*—For matched studies, give matching criteria and number of exposed and unexposed  *Case-control study*—For matched studies, give matching criteria and the number of controls per case | n.a. |
| Variables | 7 | Clearly define all outcomes, exposures, predictors, potential confounders, and effect modifiers. Give diagnostic criteria, if applicable | Methods – Paragraphs 4­–5, Tables S1, S3, S4 |
| Data sources/ measurement | 8* | For each variable of interest, give sources of data and details of methods of assessment (measurement). Describe comparability of assessment methods if there is more than one group | Methods – Paragraphs 4–7, |
| Bias | 9 | Describe any efforts to address potential sources of bias | Methods – Paragraphs 6­–7 |
| Study size | 10 | Explain how the study size was arrived at | Methods – Paragraph 2 |
| Quantitative variables  ***Table S9*** *– continued* | 11 | Explain how quantitative variables were handled in the analyses. If applicable, describe which groupings were chosen and why | Methods – Paragraphs 6-7 |
| Statistical methods | 12 | (*a*) Describe all statistical methods, including those used to control for confounding | Methods – Paragraphs 6–7, Table S2 |
|  |  | (*b*) Describe any methods used to examine subgroups and interactions | Methods – Paragraph 7, Tables S3, S4 |
|  |  | (*c*) Explain how missing data were addressed | n.a. |
|  |  | (*d*) *Cohort study*—If applicable, explain how loss to follow-up was addressed  *Case-control study*—If applicable, explain how matching of cases and controls was addressed  *Cross-sectional study*—If applicable, describe analytical methods taking account of sampling strategy | n.a. |
|  |  | (*e*) Describe any sensitivity analyses | Methods – Paragraph 7 |
| **Results** |  |  |  |
| Participants | 13* | (a) Report numbers of individuals at each stage of study—eg numbers potentially eligible, examined for eligibility, confirmed eligible, included in the study, completing follow-up, and analysed | Results – Paragraph 1, Tables 1, 2 |
|  |  | (b) Give reasons for non-participation at each stage | n.a. |
|  |  | (c) Consider use of a flow diagram | n.a. |
| Descriptive data | 14* | (a) Give characteristics of study participants (eg demographic, clinical, social) and information on exposures and potential confounders | Results – Paragraph 1, Table 1 |
|  |  | (b) Indicate number of participants with missing data for each variable of interest | n.a. |
|  |  | (c) *Cohort study*—Summarise follow-up time (eg, average and total amount) | n.a. |
| Outcome data  ***Table S9*** *– continued* | 15* | *Cohort study*—Report numbers of outcome events or summary measures over time  *Case-control study—*Report numbers in each exposure category, or summary measures of exposure  *Cross-sectional study—*Report numbers of outcome events or summary measures | Results – Paragraphs 2–4, Table 2 |
| Main results | 16 | (*a*) Give unadjusted estimates and, if applicable, confounder-adjusted estimates and their precision (eg, 95% confidence interval). Make clear which confounders were adjusted for and why they were included | Table 2 (adjusted), Table S7 (unadjusted) |
|  |  | (*b*) Report category boundaries when continuous variables were categorized | Table 1 |
|  |  | (*c*) If relevant, consider translating estimates of relative risk into absolute risk for a meaningful time period | n.a. |
| Other analyses | 17 | Report other analyses done—eg analyses of subgroups and interactions, and sensitivity analyses | Results – Paragraphs 5–8 |
| **Discussion** |  |  |  |
| Key results | 18 | Summarise key results with reference to study objectives | Discussion – Paragraph 1 |
| Limitations | 19 | Discuss limitations of the study, taking into account sources of potential bias or imprecision. Discuss both direction and magnitude of any potential bias | Discussion – Paragraphs 2, 3, 8, and 9 |
| Interpretation | 20 | Give a cautious overall interpretation of results considering objectives, limitations, multiplicity of analyses, results from similar studies, and other relevant evidence | Discussion – Paragraphs 2, 3, 4, 5, and 6 |
| Generalisability | 21 | Discuss the generalisability (external validity) of the study results | Discussion – Paragraphs 2, 6, 7, and 8 |
| **Other information** |  |  |  |
| Funding | 22 | Give the source of funding and the role of the funders for the present study and, if applicable, for the original study on which the present article is based | Section “Funding” |
